# A novel humanized Frizzled-7-targeting antibody enhances antitumor effects of Bevacizumab against triple-negative breast cancer via blocking Wnt/β-catenin signaling pathway

**DOI:** 10.1186/s13046-020-01800-x

**Published:** 2021-01-12

**Authors:** Wei Xie, Huijie Zhao, Fengxian Wang, Yiyun Wang, Yuan He, Tong Wang, Kunchi Zhang, Hao Yang, Zhaoli Zhou, Haibin Shi, Jin Wang, Gang Huang

**Affiliations:** 1 Shanghai Key Laboratory of Molecular Imaging, Shanghai University of Medicine and Health Sciences, 279 Zhouzhu Highway, Pudong New Area, Shanghai, China; 2 School of Pharmacy, Shanghai University of Medicine and Health Sciences, Shanghai, 201318 People’s Republic of China; 3 Department of Basic Medicine, School of Basic Medicine and Clinical Pharmacy, China Pharmaceutical University, Nanjing, 211198 People’s Republic of China; 4 State Key Laboratory of Radiation Medicine and Protection, School for Radiological and Interdisciplinary Sciences (RAD-X) and Collaborative Innovation Center of Radiation Medicine of Jiangsu Higher Education Institutions, Soochow University, Suzhou, 215123 People’s Republic of China

**Keywords:** Anti-Fzd7 antibody, Wnt/β-catenin pathway, TNBC, Anti-angiogenesis, Hypoxia

## Abstract

**Background:**

Anti-angiogenic therapy has been widely applied to the clinical treatment of malignant tumors. However, the efficacy of such treatments has been called into question, especially in triple-negative breast cancer (TNBC). Bevacizumab, the first anti-angiogenic agent approved by FDA, actually increases invasive and metastatic properties of TNBC cells, resulting from the activation of Wnt/β-catenin signaling in response to hypoxia. As a critical receptor of Wnt/β-catenin signaling, Frizzled-7 (Fzd7) is aberrantly expressed in TNBC, indicating Fzd7 a potential target for developing drugs to be combined with anti-angiogenic agents.

**Methods:**

Hybridoma technique and antibody humanization technique were utilized to generate a Fzd7-targeting antibody (SHH002-hu1). Biolayer interferometry (BLI) assay and near infrared (NIR) imaging were conducted to detect the affinity and targeting ability of SHH002-hu1. Next, whether SHH002-hu1 could suppress the invasion and migration of TNBC cells induced by Bevacizumab were validated, and the underlying molecular mechanisms were elucidated by luciferase reporter and western blot assays. The nude-mice transplanted TNBC models were established to assess the anti-TNBC activities of SHH002-hu1 when combined with Bevacizumab. Then, the effects on putative TNBC stem-like cells and Wnt/β-catenin signaling were evaluated by immunofluorescence (IF). Further, the tumor-initiating and self-renew capacity of TNBC cells were studied by secondary nude mouse xenograft model and sphere formation assay. In addition, the effects of SHH002-hu1 on the adaptation of TNBC cells to hypoxia were evaluated by the detection of vasculogenic mimicry (VM) and hypoxia-inducible factor-1α (HIF-1α) transcriptional activity.

**Results:**

The novel humanized antibody targeting Fzd7 (SHH002-hu1) exhibited extremely high affinity with Fzd7, and specifically targeted to Fzd7^+^ cells and tumor tissues. SHH002-hu1 repressed invasion, migration and epithelial-mesenchymal cell transformation (EMT) of TNBC cells induced by Bevacizumab through abating Wnt/β-catenin signaling. SHH002-hu1 significantly enhanced the capacity of Bevacizumab to inhibit the growth of TNBC via reducing the subpopulation of putative TNBC stem-like cells, further attenuating Bevacizumab-enhanced tumor-initiating and self-renew capacity of TNBC cells. Moreover, SHH002-hu1 effectively restrained the adaptation of TNBC cells to hypoxia via disrupting Wnt/β-catenin signaling.

**Conclusion:**

SHH002-hu1 significantly enhances the anti-TNBC capacity of Bevacizumab, and shows the potential of preventing TNBC recurrence, suggesting SHH002-hu1 a good candidate for the synergistic therapy together with Bevacizumab.

**Supplementary Information:**

The online version contains supplementary material available at 10.1186/s13046-020-01800-x.

## Background

Neovascularization has been demonstrated to be a critical factor in tumor progression and metastasis, and also the path through which tumor transition from “dormancy” to malignant [1]. Blocking angiogenesis starves tumors by depriving them of their blood supply, anti-angiogenic agents have been developed for clinical applications, and hundreds of thousands of patients have benefitted from anti-angiogenic therapy [2, 3]. However, clinical and preclinical observations indicate that these therapies may have limited efficacy [4]. Bevacizumab is a recombinant humanized monoclonal antibody that blocks angiogenesis by inhibiting vascular endothelial growth factor A (VEGFA), and FDA has approved its application for the treatment of metastatic colorectal cancer (mCRC), non-small cell lung cancer (NSCLC) and advanced ovarian cancer combined with the chemotherapy drugs. The results from the clinical trial (clinical trial number NCT-00528567) for TNBC showed that the combination of Bevacizumab and cytotoxic chemotherapy increased objective response rate (ORR) and progression-free survival (PFS) vs. chemotherapy alone, however the benefit of overall survival (OS) was not enough to support its further application [5, 6]. Emerging evidence indicated that in certain experimental conditions, anti-angiogenic agents actually increased invasive and metastatic properties of breast cancer cells [7]. Further study demonstrated that Bevacizumab treatment increased the population of cancer stem cells (CSCs) by generating intratumoral hypoxia in TNBC xenografts [4]. Over the last decade, there has been increasing evidence for the critical role that HIF-1 and Wnt/β-catenin signaling play in the tumor adaptation to anti-angiogenic therapy [3, 8], suggesting that to improve patient outcome, the anti-angiogenic agents might have to be combined with Wnt/β-catenin signaling targeting drugs or inhibitors of tumor hypoxic adaptation. Additional approaches for targeting the hypoxic tumor microenvironment have been confirmed to be potential for producing synthetic lethality in combination with anti-angiogenic therapy as a future therapeutic strategy [9, 10].

Currently, TNBC still represents the most therapeutically intractable subtype as the negative expressions of estrogen receptor (ER), progesterone receptor (PR), and human epidermal growth factor receptor 2 (HER2). Sequential single-agent chemotherapy remains the standard of care for patients with metastatic TNBC, but is associated with low response rates, short PFS and drug resistance [11, 12]. It is imperative to explore novel therapeutic options to improve the treatment outcome of TNBC. A microarray analysis to compare all the signaling pathways of TNBC and non-TNBC showed that Wnt/β-catenin signaling pathway was significantly overexpressed in TNBC. Notably, Fzd7, one of the major receptors, was up-regulated along the Wnt/β-catenin signaling pathway, and showed the greatest difference in expression as compared with other members of Fzd protein family [13, 14]. Moreover, Fzd7 has been confirmed to play a key role in stem cell biology, cancer development and progression, and always be associated with poor patient prognosis and tolerance to chemotherapy treatment [15, 16]. Hence, Fzd7 shows promise as a biomarker and a potential therapeutic target for TNBC.

In this study, we found that Bevacizumab treatment could increase the expression of Fzd7 in TNBC cells in vitro and in vivo (Supplementary Fig. 1). In vitro, Bevacizumab increased the expression of Fzd7 in MDA-MB-231/468 cells under serum deprivation condition (Supplementary Fig. 1A). Compared to TNBC tumor tissues from control animals, which from Bevacizumab-treated mice displayed significantly more areas of intense hypoxia and more expression of Fzd7; furthermore, Fzd7 positivity was found specific to zones of low oxygen (Supplementary Fig. 1B). Hence, Fzd7 was speculated to be up-regulated by Bevacizumab-induced hypoxia in vivo. Based on the above, Fzd7 is considered as a potential target for developing drugs to be combined with anti-angiogenic agents in the treatment of TNBC.

Here, a novel humanized anti-Fzd7 antibody (SHH002-hu1) with high affinity was generated, and its specificity and targeting ability were verified. Subsequently, the anti-TNBC effects of SHH002-hu1 in vitro were evaluated, and the role SHH002-hu1 played in Wnt/β-catenin signaling was investigated. Then, the effects of SHH002-hu1 on Bevacizumab-induced (EMT) in MDA-MB-231/MDA-MB-468 cells were studied in vitro, and the relevant mechanism was elucidated. In addition, whether SHH002-hu1 could enhance the capacity of Bevacizumab to inhibit MDA-MB-231/MDA-MB-468 tumor growth was validated, and the predicted reversal effects of SHH002-hu1 on the increased subpopulation of putative TNBC stem-like cells induced by Bevacizumab were confirmed. The potential of SHH002-hu1 to attenuate the self-renew and tumor-initiating capacity of TNBC cells enhanced by Bevacizumab was further studied. Through the above-mentioned studies, we hope to provide a potential targeted agent which could enhance the anti-TNBC effects of Bevacizumab, and lay foundation for the synergistic strategy of combining anti-angiogenic agents with Fzd7-targeting agent.

## Materials and methods

### Cell culture

The Chinese hamster ovary cell line CHO-s (Sanyou, Shanghai, China) and human mammary epithelial cell line MCF-10A (American Type Culture Collection, New York, USA) were maintained in DMEM/F12 medium (Gibco, Grand Island, USA), supplemented with 10% (v/v) FBS (Gibco, Auckland, NZ). The human embryonic kidney cell line HEK293T kept in our laboratory was cultured in DMEM high glucose medium (Gibco, New York, USA), supplemented with 10% (v/v) FBS. The human breast cancer cell lines MDA-MB-231/MDA-MB-468 (American Type Culture Collection) were maintained in L-15 medium (Gibco, New York, USA) supplemented with 10% (v/v) FBS. HUVECs (ScienCell, San Diego, CA) were maintained in endothelial culture medium (ECM, ScienCell) supplemented with 5% (v/v) FBS and 1% (v/v) endothelial cell growth supplement (ECGS, ScienCell). All cells were maintained at 37 °C in a humidified atmosphere with 5% CO_2_.

### Binding affinity and kinetic analysis

SHH002-hu1 was expressed in CHO-s cells and purified by Protein G affinity chromatograph (GE Healthcare, Buckinghamshire, UK), followed by the analysis of SDS-PAGE and SEC-HPLC. The binding kinetics of SHH002-hu1 to rhFzd1/2/5/7/8 was measured with Fortebio Octet Red96 (PALL, USA). Firstly, SHH002-hu1 was captured by the Anti-Human Fab-CH1 2nd Generation Sensor (PALL, USA). Then, rhFzd1/2/5/7/8 was injected at different concentrations into running buffer (KB buffer: 0.1% BSA + 0.05% Tween 20 dissolved in PBS, pH 7.2), and capture was done at a constant flow rate. As for SHH002 (the murine antibody targeting Fzd7 generated by us), the antibody was captured by the Anti-Biotin Sensor (PALL, USA) after conjugated with biotin. Sensorgrams were obtained at each concentration, and the association rate constant (*ka*) and dissociation rate constant (*kd*) were calculated by the instrument algorithm. Finally, the equilibrium dissociation constant (*KD*) was calculated from the ratio of rate constants *kd*/*ka*.

### IF assay for the binding of SHH002-hu1

The gene of human Fzd7 was linked with the lentiviral vector (HBLV-GFP-PURO, Hanbio Biotechnology, China) to obtain the recombinant plasmid HBLV-h-Fzd7–3*flag-GFP-PURO. HEK293T cells stably overexpressing Fzd7 (Fzd7 OE) were obtained through virus infection and screening. HEK293T cells (blank/vector control/Fzd7 OE), MDA-MB-231/MDA-MB-468 cells were inoculated into 6-well plate. When cell confluence reached 70–80%, the cells were incubated with SHH002-hu1 and then Goat Anti-Human IgG H&L (Alexa Fluor 647, Abcam, USA). Subsequently, images were taken by an OLYMPUS fluorescence microscope (Olympus, Tokyo, Japan).

### In vivo dynamics and targeting capability by NIR imaging

5-week-old female BALB/c-nude mice were purchased from Shanghai Lab. Animal Research Center, China. 1 × 10^7^ MDA-MB-231 cells were injected into the mammary fat pads of mice. When the average tumor volume reached 100 mm^3^, mice were randomized into 2 groups (*n* = 5 for each group). IRB-NHS fluorescence probing (Keyuandi Biotechnology, Shanghai, China) was incubated with SHH002-hu1 for 2 h to form NIRB-SHH002-hu1 fluorescence probe. NIRB-SHH002-hu1 (50 nmol/kg) was then intravenously injected into TNBC tumor-bearing mice. Additionally, free SHH002-hu1 (2.5 μmol/kg) was mixed with NIRB-SHH002-hu1 (50 nmol/kg) to evaluate the competitive blocking. After intravenous injection, fluorescence images were taken by IVIS Spectrum CT imaging system (PerkinElmer, USA) at different time intervals. The tumor/normal tissue ratios were analyzed from the region of interests (ROI).

### Cell proliferation assay

4 × 10^3^ HEK293T/MCF-10A/MDA-MB-231/MDA-MB-468 cells were seeded into a 96-well plate to attach. Then, different concentrations of SHH002-hu1 were added and pre-incubated for 2 h, after which recombinant Wnt3a (R&D, Minneapolis, MN, USA) was added at a final concentration of 200 ng/mL. The SHH002-hu1 untreated group with Wnt3a induced was as vehicle control. After incubation for 48 h, cell viability was quantified by MTT assay and the proliferation rate was expressed as percentage of the vehicle control (100%).

### Apoptosis assays

5 × 10^5^ MDA-MB-231/MDA-MB-468 cells were seeded in 6-well plates and allowed to adhere. When reaching 70% confluence, cells were incubated with 100 nM SHH002-hu1 for 48 h. Then the cells were stained with Annexin V (AV)-FITC and propidium iodide (PI) following manufacturer’s protocol of FITC Annexin V Apoptosis Detection Kit II (BD Biosciences, USA). Finally, the data of apoptosis were detected by a Beckman CytoFLEX S flow cytometer (Beckman Coulter, USA).

### Luciferase reporter assay

The β-catenin/TCF-driven transcriptional activity was assessed by transient transfection of MDA-MB-231/MDA-MB-468 cells with the TOP-FLASH/FOP-FLASH luciferase reporter assay and a Renilla luciferase transfection control reporter. MDA-MB-231/MDA-MB-468 cells were seeded into 48-well plates and transfected with TOP-FLASH/FOP-FLASH plasmid using Lipofectamine™ 2000 (Invitrogen, Cartsbad, CA, USA). Then, the cells were treated with SHH002-hu1 (100 nmol/L)/FH535 (10 μmol/L, absin, China), 2 h later, Wnt3a (200 ng/mL) was added. The luciferase activities were measured at 24 h with the Dual-Glo luciferase assay reporter system (Promega, Madison, WI, USA).

### IF assay for the location of β-catenin

5 × 10^5^ MDA-MB-231/MDA-MB-468 cells were seeded on cover slips in 6-well plates and allowed to adhere. When reaching 70% confluence, cells were treated with SHH002-hu1 (100 nmol/L)/FH535 (10 μmol/L), 2 h later, Wnt3a (200 ng/mL) was added. After another 22 h, cells were fixed and then incubated with α-β-catenin (Cell Signaling Technology, USA) followed by FITC-conjugated secondary antibody. Images were taken by an OLYMPUS fluorescence microscope at 400-times magnification after the incubation with DAPI stain solution (Sangon Biotech, Shanghai, China).

### Western blot assay

The whole cell proteins of MDA-MB-231/MDA-MB-468 cells were extracted from cells using RIPA buffer (Beyotime, Shanghai, China) and the nuclear extracts were prepared with a NE-PER Nuclear and Cytoplasmic Extraction Kit (Pierce Biotechnology, Rockford, USA). Western blots were probed with α-phospho LRP6 (Ser1490), α-LRP6, α-β-catenin, α-E-cadherin, α-N-cadherin, α-Vimentin, α-Snail, α-Histone H3, α-c-Myc, α-cyclin D1, α-Axin, α-CD44, α-HIF-1a, α-Glut1 (Cell Signaling Technology, USA), α-VEGFA (Abcam, Cambridge, UK), α-β-actin.

### Tube formation assay

2 × 10^4^ HUVECs were seeded into the 96-well plate coated with matrigel (Corning, Bedford, USA), and added with the supernatants obtained from SHH002-hu1 treated MDA-MB-231/MDA-MB-468 cells. After 8-h incubation, endothelial tube formation was photographed with an inverted OLYMPUS microscope, 10 ng/mL VEGF_165_ (Sino Biological, Beijing, China) treated HUVECs were as control. The endothelial tubes were counted with Image-Pro-Plus program and the tube formation rate quantified on the basis of the control.

### Transwell invasion and wound healing assays

1 × 10^4^ of MDA-MB-231/MDA-MB-468 cells suspended in serum-free medium were plated into the upper wells of 24-well transwell chamber (Millipore, Billerica, USA) coated matrigel, and then treated with Bevacizumab (200 nmol/L, Roche, France)/Bevacizumab (200 nmol/L) + SHH002-hu1 (100 nmol/L)/Bevacizumab (200 nmol/L) + FH535 (10 μmol/L). The lower chambers were filled with complete medium. 12 h later, the invaded cells were then fixed and stained. Images were taken using an OLYMPUS inverted microscope. Invaded cells were counted using Image-Pro-Plus program and invasion percentages quantified on the basis of untreated control.

3 × 10^4^ MDA-MB-231/MDA-MB-468 cells were placed into each well of Culture-Insert (Ibidi, Martinsried, Germany) in 24-well plate. After adherence, the Culture-Insert was removed and Bevacizumab (200 nmol/L)/Bevacizumab (200 nmol/L) + SHH002-hu1 (100 nmol/L)/Bevacizumab (200 nmol/L) + FH535 (10 μmol/L) dissolved in serum-free medium was added into the wells. Images were taken with OLYMPUS inversion fluorescence microscope at 0, 8, 16 h after the addition of the treatments. The wound migrated distances were measured with Image-Pro-Plus program and calculated as follows: L_n_ = (L_0_-L_time_)/2.

### Cell xenografts in nude mice assay

The xenograft tumors of MDA-MB-231/MDA-MB-468 cells in nude mice were established as described above. When the average tumor volume reached 50 mm^3^, mice were randomized into 5 groups (*n* = 5 for each group), and the administration began: (1) PBS control; (2) 5 mg/kg Bevacizumab (intravenous injection, twice a week); (3) 5 mg/kg SHH002-hu1 (intravenous injection, twice a week); (4) 5 mg/kg Bevacizumab + 5 mg/kg SHH002-hu1; (5) 5 mg/kg Bevacizumab + 10 mg/kg Docetaxel (intravenous injection, every 3 days). Tumors were measured by digital calipers periodically and the tumor volume was determined as V = (length × width^2^)/2. At the end of drug treatment, the mice were humanely euthanized and tumors were harvested for further studies.

### IF and immunohistochemistry (IHC) analysis

For ALDH1/Hypoxyprobe and β-catenin/Hypoxyprobe double labeling, mice were injected intravenously with 60 mg/kg of the pimonidazole solution, 90 min later, the mice were euthanatized and tumor tissues were removed and snap-frozen. Frozen tissue sections were then interrogated with FITC-conjugated α-pimonidazole (Hypoxyprobe Inc., USA) and α-ALDH1 (Abcam, USA)/β-catenin (Cell Signaling Technology, USA) followed by respective Cy3-conjugated secondary IgG. Coverslips were then mounted with DAPI stain solution. For Periodic Acid Schiff (PAS)-CD31 double IHC staining, paraffin sections were cut into 5 μm sections and fixed in 4% paraformaldehyde. The sections were firstly incubated with α-CD31 (Cell Signaling Technology, USA), then exposed to 1% sodium periodate and incubated with PAS (BestBio, Shanghai, China).

### Secondary nude mouse xenograft model

MDA-MB-231/MDA-MB-468 tumors tissues were minced into small pieces and processed with collagenase for 2 h at 37 °C. After centrifugation, the cell pellets were trypsinized and passed through an 80-μm filter to produce single-cell suspension, then living cells were sorted out by fluorescence-activated cell sorting. Each nude mouse was inoculated with 1 × 10^4^ cells from control/Bevacizumab-treated/Bevacizumab + SHH002-hu1-treated tumors in one of the inguinal mammary fat pads. The growth of tumors was monitored and tumor sizes were measured weekly.

### Sphere formation assay

The single-cell suspension was produced as above, then 5 × 10^3^ dissociated tumor cells were seeded on ultralow attachment 6-well plates in serum-free medium DMEM/F12 (Gibco, Grand Island, USA) supplemented with B27 (Gibco, Grand Island, USA), 20 ng/mL human recombinant fibroblast growth factor (FGF), and 20 ng/mL epidermal growth factor (EGF, Sino Biological Inc., Beijing, China). The mammospheres (diameter > 60 μm) were counted under an OLYMPUS inversion fluorescence microscope.

### Statistical analysis

All data were presented as the mean ± standard deviation (SD). Differences between multiple groups were analyzed by the student’s *t* test and *p* values of 0.05 or less were considered statistically significant.

## Results

### SHH002-hu1 exhibits high affinity with rhFzd7 and targets Fzd7^+^ TNBC tumor tissues specifically

**Fig. 1:**
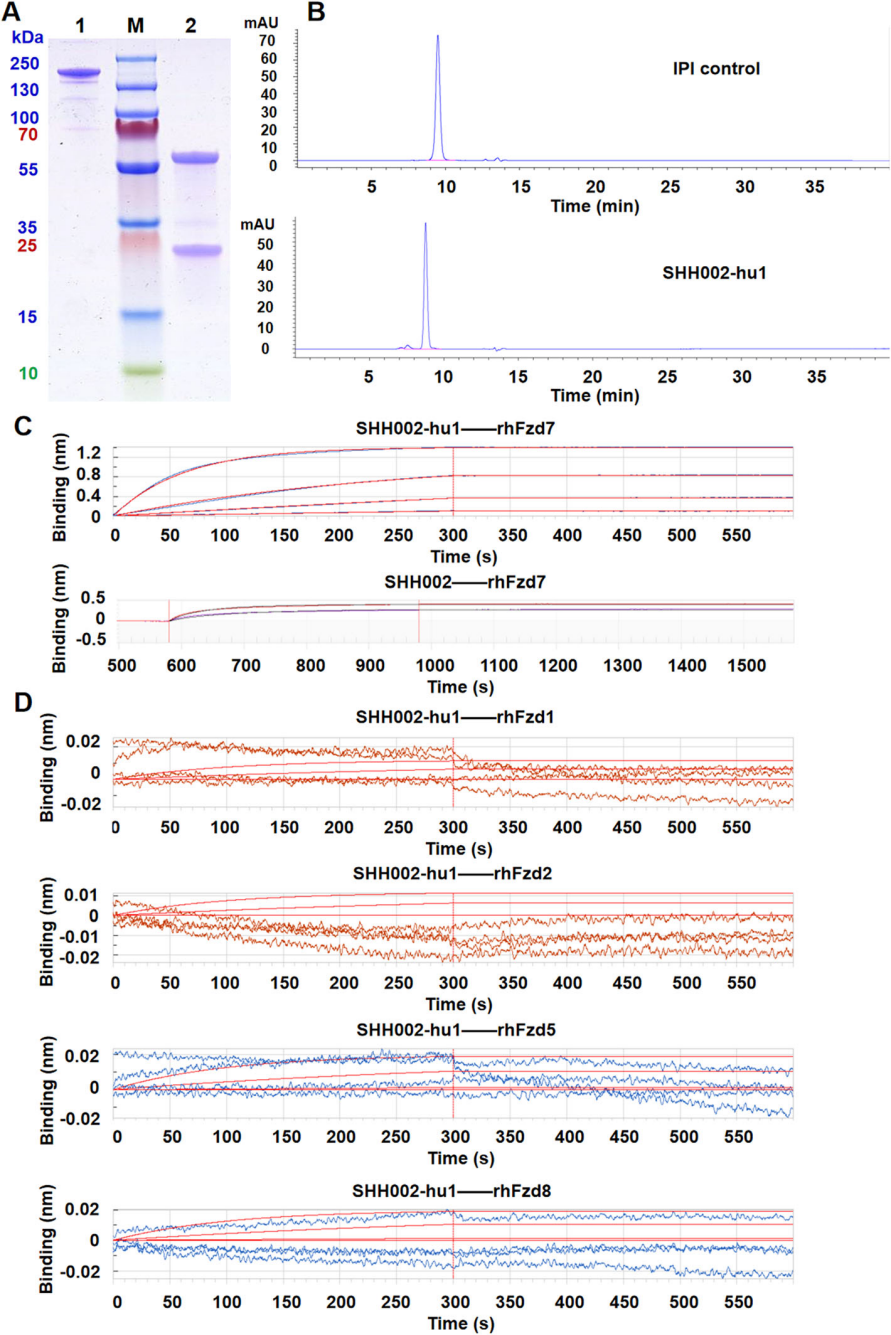
The identification and affinity detection of SHH002-hu1. **a**. SDS-PAGE analysis of SHH002-hu1. Lane 1 represented non-reducing and lane 2 represented reducing condition. Lane M: Marker. **b**. SEC-HPLC analysis of SHH002-hu1. The peak shape of IPI control/SHH002-hu1chromatogram was presented well, and the baseline was stable. The criteria of system suitability was satisfied. **c**. Set of sensorgrams of rhFzd7 binding with SHH002-hu1 and SHH002. For SHH002-hu1, the association rate increased with increasing concentration of the rhFzd7 (from bottom to top: 6.25, 25, 100, 400 nmol/L). 0 s - 300 s: association, 300 s - 600 s: dissociation. For SHH002, the association rate increased with increasing concentration of the rhFzd7 (from bottom to top: 300, 750 nmol/L). 600 s - 1000 s: association, 1000 s - 1580 s: dissociation. **d**. Set of sensorgrams of rhFzd1/rhFzd2/rhFzd5/rhFzd8 protein binding with SHH002-hu1. 0 s - 300 s: association, 300 s - 600 s: dissociation. The association rate had no correlation with the concentration of rhFzd1/2/5/8. The red smooth curves in each figure represented the Fortebio fitting curves

The amino acid sequence of the humanized antibody targeting Fzd7 (SHH002-hu1) was acquired through an elaborate humanized design from the murine antibody SHH002 (Supplementary Fig. 2). Then, SHH002-hu1 was expressed in CHO-s cells and purified by Protein G affinity chromatograph. SDS-PAGE analysis of the purified SHH002-hu1 (Fig. 1a) showed Heavy Chain (HC, 50 kDa) and Light Chain (LC, 25 kDa) of the expected molecular weight were expressed correctly, and assembled into complete antibodies of 150 kDa. SEC-HPLC analysis (Fig. 1b) demonstrated the purity of purified SHH002-hu1 was more than 95%. BLI assay (Fig. 1c) indicated that SHH002-hu1 exhibited extremely high affinity with rhFzd7 (*ka* (1/Ms): (4.00 ± 0.32) × 10^5^, *kd* (1/s) < 1.0 × 10^− 7^, *KD* (M) < 1.0 × 10^− 12^, *n* = 3), similar to that of SHH002 (*ka* (1/Ms): (2.85 ± 0.27) × 10^5^, *kd* (1/s) < 1.0 × 10^− 7^, *KD* (M) < 1.0 × 10^− 12^, n = 3). To confirm the binding specificity of SHH002-hu1, BLI assay was carried out and the results (Fig. 1d) indicated that SHH002-hu1 showed no cross-reactions with rhFzd1/2/5/8 (Fzd protein family members Fzd1, Fzd2, Fzd5, Fzd8 share high homology with Fzd7).

**Fig. 2:**
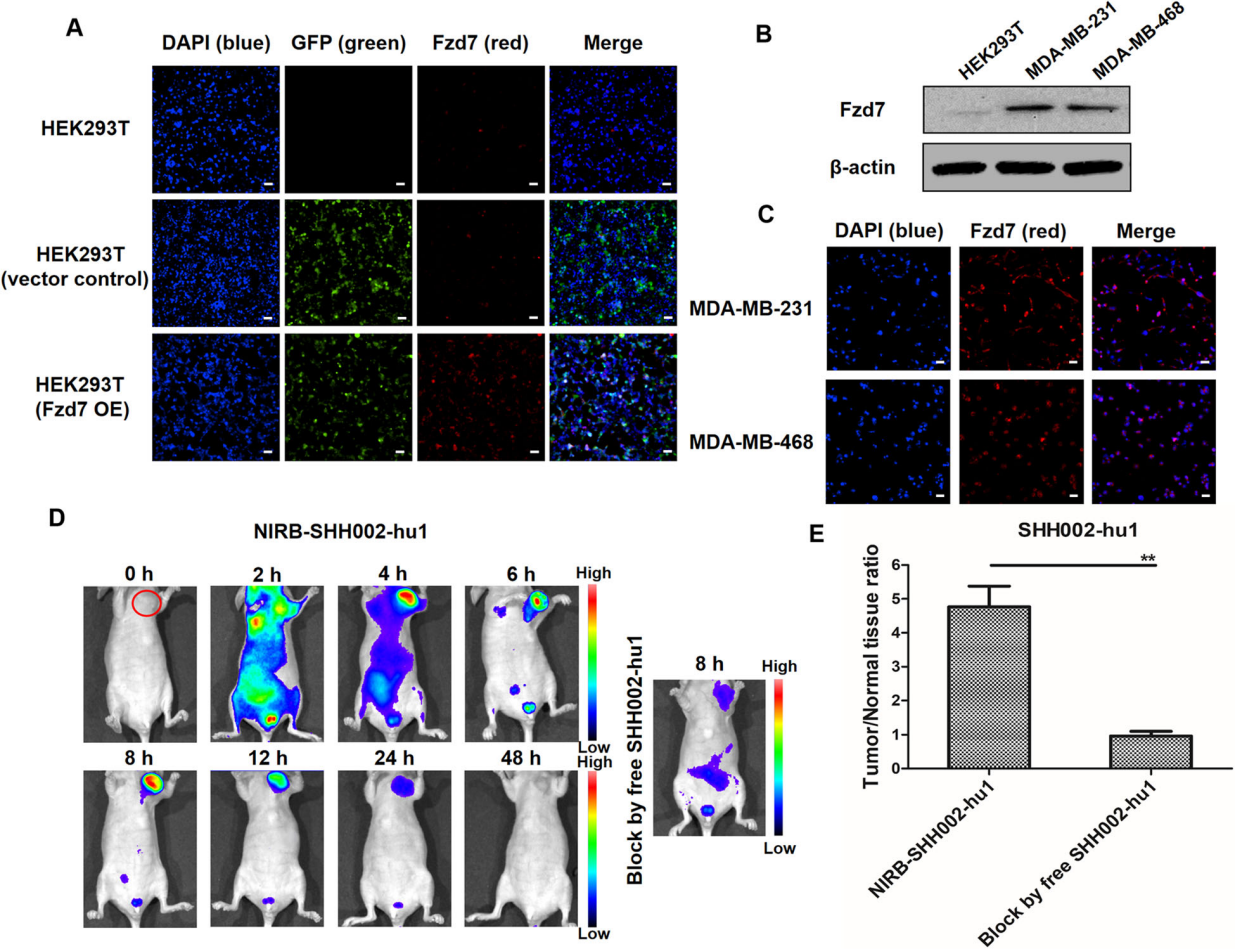
SHH002-hu1 specifically targets Fzd7^+^ cells and Fzd7^+^ TNBC tumor tissue. **a**. Characterization of SHH002-hu1 by immunofluorescent staining of transfected cells. SHH002-hu1 showed strong binding signal (red) to Fzd7 overexpressing HEK293T cells, HEK293T cells not transfected or transfected with empty vector (GFP label) were used as control. Bar = 50 μm. **b**. Western blot assay to detect the expression of Fzd7 in TNBC cells. The detection antibody was Rabbit Anti-Human Fzd7 purchased from Abcam. **c**. IF assay to detect the binding of SHH002-hu1 with Fzd7 high-expressing MDA-MB-231/MDA-MB-468 cells. Bar = 50 μm. **d**. The bio-distribution of NIRB-SHH002-hu1 was evaluated by the NIRB imaging assay in MDA-MB-231-bearing nude mice. In blocking experiments, free SHH002-hu1 inhibited the probes from binding to the tumor sites. **e**. Tumor/normal tissue ratios calculated at 8 h post-injection of probe groups into MDA-MB-231-bearing nude mice from the region of interest. Data were given as the mean ± SD (*n* = 5), ^**^p < 0.01

IF assay (Fig. 2a) demonstrated that SHH002-hu1 effectively bound to Fzd7-overexpressing HEK293T cells rather than the blank HEK293T cells. The obvious binding of SHH002-hu1 to Fzd7 high-expressing TNBC cells (Fig. 2b) was observed (Fig. 2c). Subsequently, NIR imaging was used to assess the dynamics and targeting capability of NIRB-SHH002-hu1 in vivo. After the injection of NIRB-SHH002-hu1, fluorescent signals spread throughout the body immediately. 4 h later, some fluorescent antibody was excreted through the kidneys, and MDA-MB-231 xenografts were distinguished by fluorescence. The fluorescence signal was maintained for 12 h, and a weak signal could still be detected at 24 h (Fig. 2d). At the meantime, the blocking group showed no intense fluorescent signals at the tumor site, indicating that tumor targeting is mediated by SHH002-hu1. The fluorescent signals were significantly different, with a maximal tumor/normal tissue ratio at 8 h of 4.77 ± 0.87 and 0.96 ± 0.35 for the NIRB-SHH002-hu1 treated group and its blocking group, respectively (Fig. 2e). Consequently, SHH002-hu1 effectively targets Fzd7^+^ cells and Fzd7^+^ TNBC tumor tissues by specifically binding to Fzd7.

### SHH002-hu1 inhibits the proliferation of TNBC cell lines and tumor angiogenesis through blocking Wnt/β-catenin pathway

**Fig. 3:**
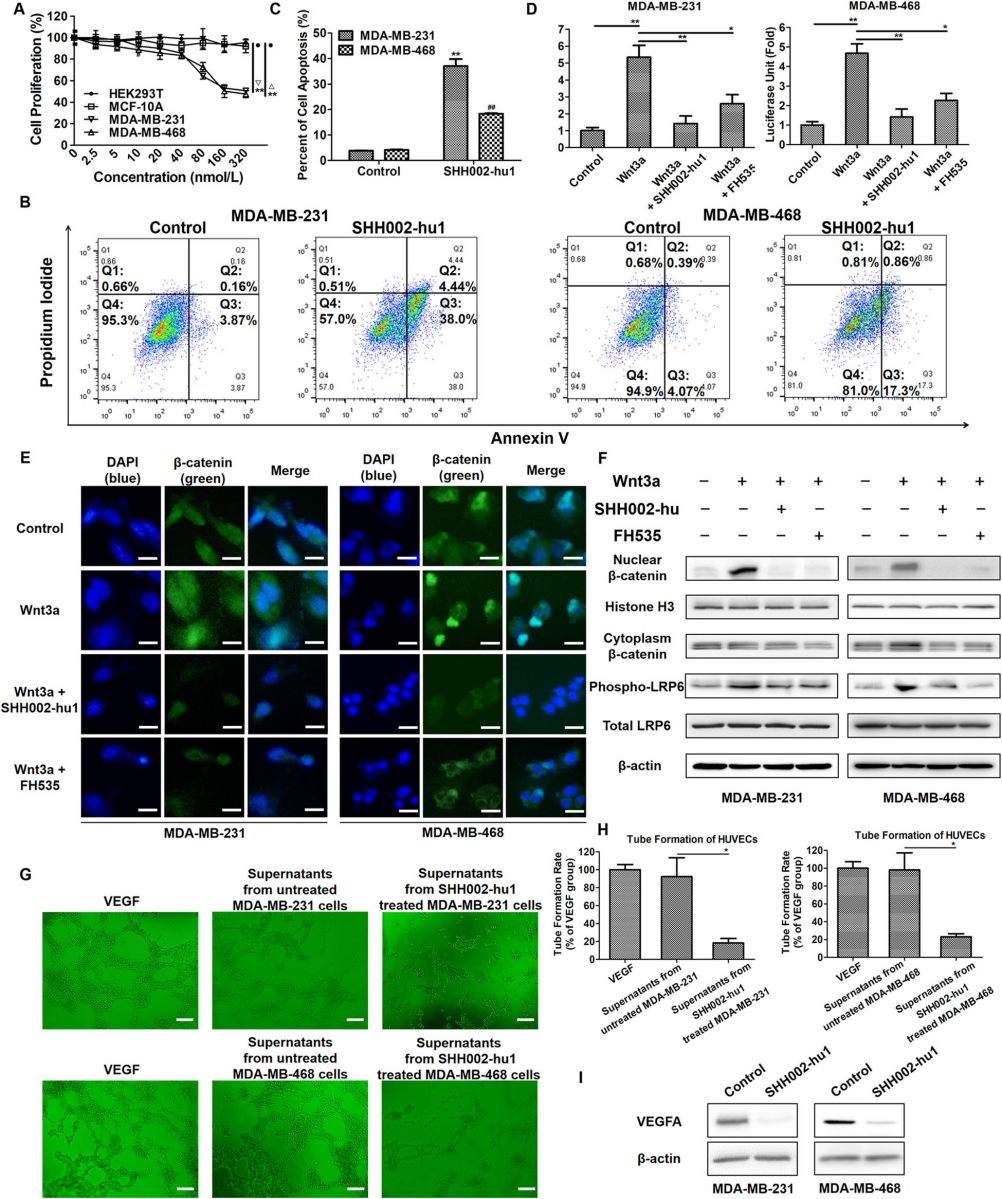
SHH002-hu1 inhibits the proliferation of TNBC cell lines and tumor angiogenesis through blocking Wnt/β-catenin pathway. **a**. The viability of MDA-MB-231/MDA-MB-468 cells was assessed by MTT assay at 48 h after treatment with different concentrations of SHH002-hu1. HEK293T cell and MCF-10A were set as a negative control. SHH002-hu1 specifically inhibited the growth of Fzd7^+^ TNBC cells induced by Wnt3a in a dose-dependent manner. Data were given as the mean ± SD (*n* = 3), ^**^p < 0.01. **b**. Representative plots showing the apoptosis patterns of SHH002-hu1 treated MDA-MB-231/MDA-MB-468 cells, the percentage of cells in each quadrant was indicated. **c**. Quantitative analysis of apoptosis assay. Data were presented as the mean ± SD, n = 3, ^**^p < 0.01 (MDA-MB-231 cells), vs. the previous group; ^##^*p* < 0.01 (MDA-MB-468 cells), vs. the previous group. **d**. TOP/FOP ratio in MDA-MB-231/MDA-MB-468 cells (stimulated by Wnt3a) treated with SHH002-hu1 for 24 h. FH535 was set as a positive control. The results from 3 independent experiments are expressed as mean ± SD of fold change, ^*^p < 0.05, ^**^p < 0.01. SHH002-hu1 effectively inhibited the β-catenin/TCF-4 transcriptional activity induced by Wnt3a. **e**. SHH002-hu1 repressed the accumulation of β-catenin in the nucleus induced by Wnt3a. IF stainings of β-catenin (green) were shown, and nuclei were counterstained with DAPI (blue), bar = 20 μm. **f**. SHH002-hu1 blocked nuclear accumulation of β-catenin and phosphorylation of LRP6. MDA-MB-231/MDA-MB-468 cells were treated with Wnt3a and SHH002-hu1, then western blot assay was conducted as indicated. Histone H3 was used as loading control for nuclear proteins, and β-actin was for cytoplasmic proteins. SHH002-hu1 attenuated the Wnt3a induced accumulation of β-catenin and inhibited the induction of phosphorylated (Ser1490) LRP6 by Wnt3a. **g**, **h**. HUVECs tube-like photomicrographs and quantitative analysis revealed that the angiogenesis in in vitro model was significantly inhibited by the 8 h-incubation of SHH002-hu1-treated TNBC cells supernatants, bar = 100 μm. The quantitative analysis of HUVECs tube formation (a complete polygon was considered as a tube) was based on the mean of the 5 regions of each group. Data were presented as the mean ± SD, *n* = 5, ^*^p < 0.05. **i**. Western blot assay indicated that SHH002-hu1 reduced the expression of VEGFA in TNBC cells remarkably

MTT assay showed that SHH002-hu1 effectively inhibited the proliferation of MDA-MB-231/MDA-MB-468 cells in a dose-dependent manner (Fig. 3a), the maximum inhibitory rate was (49.26 ± 1.25)% for MDA-MB-231 cells and (52.33 ± 3.21)% for MDA-MB-468 cells respectively. Whereas SHH002-hu1 showed no significant effect on the proliferation of HEK293T cells and MCF-10A (normal breast epithelial) cells, indicating the specifically antiproliferative activity of SHH002-hu1 towards TNBC tumor cells. Then, apoptosis assay was executed. After the treatment of SHH002-hu1, the proportion of MDA-MB-231/MDA-MB-468 cells in Q2 and Q3 quadrants increased significantly (Fig. 3b, c), indicating the apoptosis-inducing function of SHH002-hu1. TOP-FLASH/FOP-FLASH luciferase reporter assay for Wnt/β-catenin downstream TCF/LEF transcription activity was used to measure Wnt/β-catenin functions. SHH002-hu1 obviously decreased the transcriptional activity of TCF/LEF enhanced by Wnt3a, indicating the inhibition of Wnt/β-catenin signaling (Fig. 3d). Since the transcriptional activity of β-catenin required its translocation from the cytoplasm to the nucleus, IF assay was performed to evaluate the distribution pattern of β-catenin. Compared to the control, Wnt3a promoted the translocation of β-catenin from the cytoplasm to the nucleus in TNBC cells. SHH002-hu1 could strongly suppress the nuclear β-catenin accumulation induced by Wnt3a, similar to FH535 (a small-molecule inhibitor of Wnt/β-catenin signaling pathway) (Fig. 3e). Western blot assay indicated that SHH002-hu1 reduced both Wnt3a-induced nuclear accumulation of β-catenin and phosphorylation of LRP6 significantly (Fig. 3f). The shRNA of Fzd7 (h-Fzd7 shRNA) was also utilized in the IF and Western blot assay as a positive control of Fzd7 inhibitor (Supplementary Fig. 3). As Wnt/β-catenin pathway activity positively correlated with elevated angiogenesis in TNBC, we further investigated whether SHH002-hu1 could repress angiogenesis in a TNBC model in vitro. The HUVEC tube formation potency of supernatant obtained from SHH002-hu1-treated MDA-MB-231/MDA-MB-468 cells was significantly reduced (Fig. 3g, h). Western blot assay (Fig. 3i) showed that SHH002-hu1 inhibited the expression of VEGFA in TNBC cells. Therefore, SHH002-hu1 could repress TNBC angiogenesis effectively in vitro. Supplementary Fig. 4 indicated the schematic representation of a model wherein SHH002-hu1 inhibited Wnt/β-catenin signaling pathway via blocking the ability of Wnt proteins to interact with Fzd7.

### SHH002-hu1 suppresses Bevacizumab-induced transwell, migration and EMT of TNBC cells via abating Wnt/β-catenin pathway

**Fig. 4:**
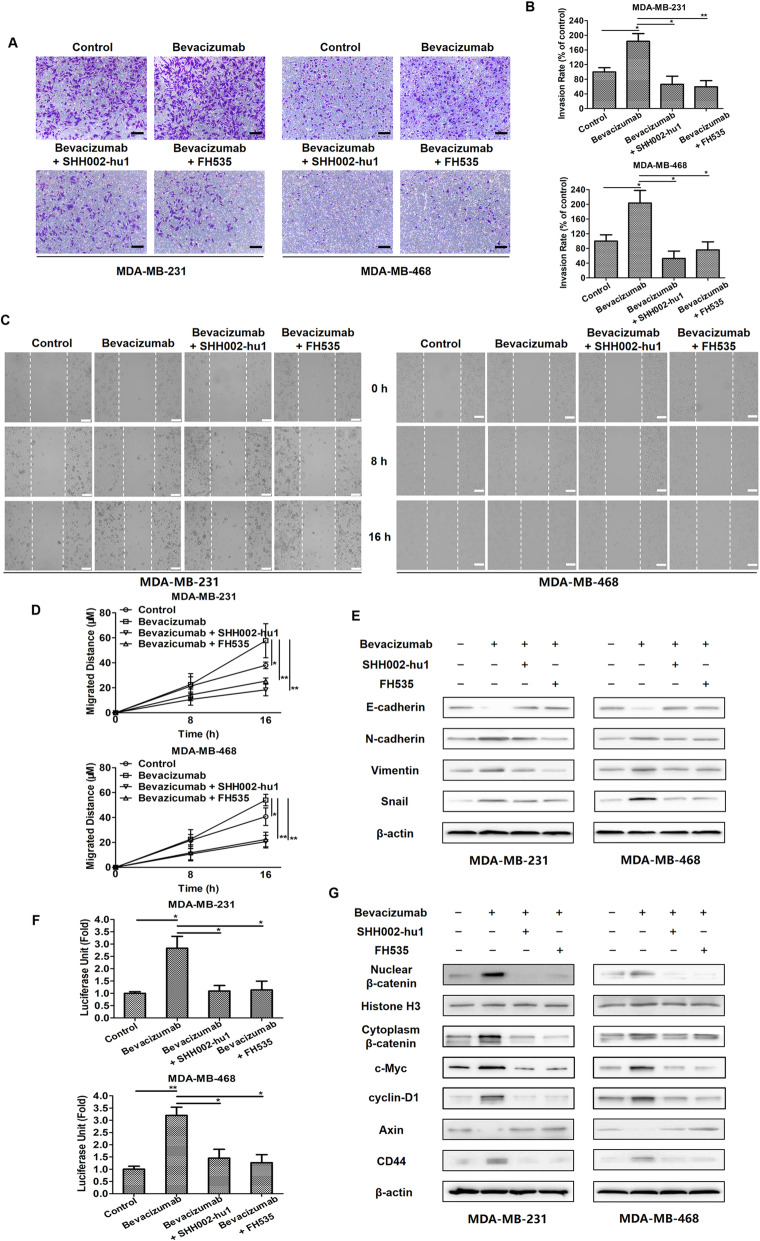
SHH002-hu1 inhibits Bevacizumab-induced transwell, migration and EMT of TNBC cells via abating Wnt/β-catenin pathway. **a**. Microscopic views from transwell assay to estimate MDA-MB-231/MDA-MB-468 cells invasion following 24 h treatment of Bevacizumab/Bevacizumab + SHH002-hu1/Bevacizumab + FH535, bar = 100 μm. **b**. Quantitative analysis of (**a**) by Image J. **c**. Photomicrographs of cell migration from wound healing assay in MDA-MB-231/MDA-MB-468 cells treated with Bevacizumab/Bevacizumab + SHH002-hu1/Bevacizumab + FH535, bar = 100 μm. **d**. Quantitative analysis of (**c**) by Image J. The quantitative analyses of transwell invasion assay and wound healing assay were based on the mean of the 5 regions of each group. Data were presented as the mean ± SD, n = 5, ^*^p < 0.05, ^**^p < 0.01. **e**. SHH002-hu1 inhibited Bevacizumab-induced EMT in TNBC cells. MDA-MB-231/MDA-MB-468 cells were incubated with Bevacizumab/Bevacizumab + SHH002-hu1/Bevacizumab + FH535 for 24 h. Then the results of western blot analysis for EMT marker proteins were shown. **f**. TOP/FOP ratio in MDA-MB-231/MDA-MB-468 cells treated for 24 h with Bevacizumab/Bevacizumab + SHH002-hu1/Bevacizumab + FH535. SHH002-hu1/FH535 inhibited the β-catenin/TCF-4 transcriptional activity induced by Bevacizumab significantly. Data were presented as the mean ± SD, *n* = 3, ^*^p < 0.05, ^**^p < 0.01. **g**. SHH002-hu1 suppressed the activation of Wnt/β-catenin pathway induced by Bevacizumab. The results of western blot analysis for the expression of β-catenin and downstream oncoproteins were shown

Bevacizumab, as well as some other anti-angiogenic agents, has been recently demonstrated to be able to facilitate the migration and invasion of breast cancer cells through activating Wnt/β-catenin signaling pathway excessively [4]. Here, transwell invasion and wound healing assays were performed for the evaluation of MDA-MB-231/MDA-MB-468 cell migration. Following Bevacizumab treatment under serum starvation, increased cell invasion (Fig. 4a, b) and migration (Fig. 4c, d) were detected. As increased invasion and migration are outcomes of the EMT process, which indicated Bevacizumab could induce EMT of TNBC cells in the serum-deprived environment. Further, the detection of the expression level of various classical EMT markers in TNBC cells (Fig. 4e) confirmed the above speculation. The enhanced transcriptional activity of TCF/LEF (Fig. 4f) and nuclear accumulation of β-catenin (Fig. 4g) induced by Bevacizumab indicated that Bevacizumab could significantly activate Wnt/β-catenin pathway, and Bevacizumab-induced EMT involved the activation of Wnt/β-catenin pathway.

Fzd7, as a potential therapeutic target for TNBC, is verified to be up-regulated along the Wnt/β-catenin signaling pathway [13, 14]. As shown in Supplementary Fig. 1A, B, Bevacizumab treatment could increase the expression of Fzd7 both in vitro and in vivo, probably resulting from the abnormally activated Wnt/β-catenin pathway. Through targeting Fzd7 specifically, SHH002-hu1 potently inhibited the transcriptional activity of TCF/LEF (Fig. 4f) and nuclear accumulation of β-catenin induced by Bevacizumab (Fig. 4g), resulting in the remarkably suppressed TNBC cell invasion (Fig. 4a) and migration (Fig. 4c). Likewise, the stimulated EMT of MDA-MB-231/MDA-MB-468 cells induced by Bevacizumab was weakened along with the attenuated Wnt/β-catenin pathway (Fig. 4e, g).

### SHH002-hu1 significantly enhances the capacity of Bevacizumab to inhibit MDA-MB-231/MDA-MB-468 tumor growth, and reduces the percent of ALDH1^+^ TNBC cells increased by Bevacizumab

Based on the above analysis, SHH002-hu1 could suppress Wnt/β-catenin pathway and inhibit EMT induced by Bevacizumab effectively under serum starvation condition in vitro. It has been verified that the anti-angiogenic agents Sunitinib and Bevacizumab increase the population of CSCs by generating intratumoral hypoxia and activating Wnt/β-catenin signaling pathway in TNBC xenografts [4], causing the increased invasive and metastatic properties of tumor cells. Hence, the efficacy of anti-angiogenic agents against TNBC was greatly limited. Here, TNBC cell xenografts in nude mice assay were executed to investigate whether SHH002-hu1 could enhance the anti-tumor effect of Bevacizumab in vivo via reversing the increased population of CSCs.

**Fig. 5:**
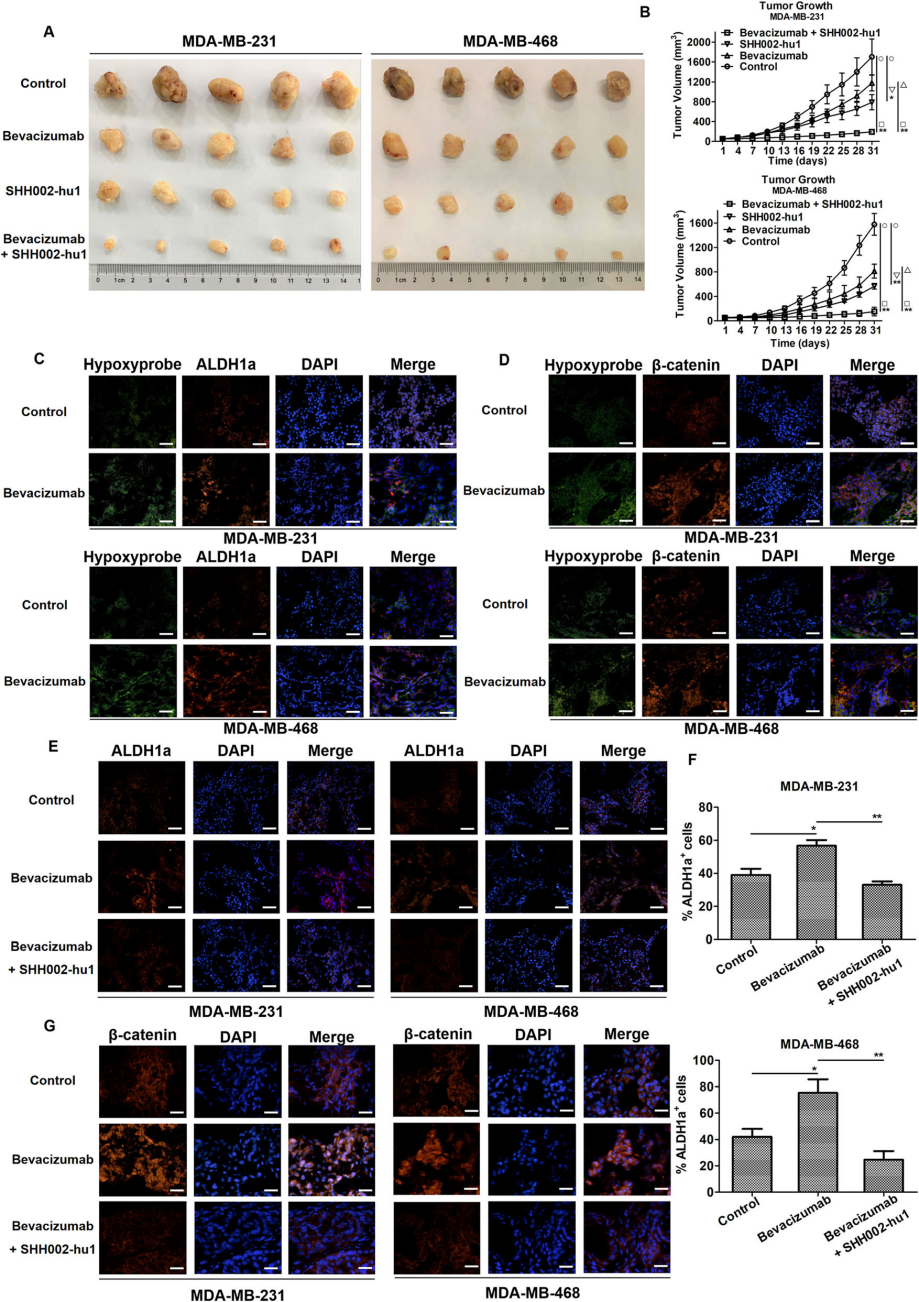
SHH002-hu1 enhances the ability of Bevacizumab to inhibit MDA-MB-231/MDA-MB-468 tumor growth significantly. **a**. Representative images of isolated tumors from MDA-MB-231 (the left 5 rows)/MDA-MB-468 (the right 5 rows)-tumor bearing nude mice. **b**. MDA-MB-231/MDA-MB-468 tumor growth curves of each group under different treatments. Data were given as the mean ± SD (n = 5), ^*^p < 0.05, ^**^p < 0.01. **c**. Bevacizumab induced hypoxia in TNBC tumor tissues in vivo, and ALDH1^+^ cells were concentrated in hypoxic regions. Hypoxia in MDA-MB-231/MDA-MB-468 tumors was detected by IF staining of pimonidazole adducts in sections from different groups. Staining showed pimonidazole immunodetection (green) and ALDH1 (red) merged with DAPI-stained nuclei (blue), bar = 100 μm. **d**. The β-catenin pathway of MDA-MB-231/MDA-MB-468 tumor was stimulated in response to hypoxia after the treatment of Bevacizumab. Staining showed pimonidazole immunodetection (green) and β-catenin (red) merged with DAPI-stained nuclei (blue), bar = 100 μm. **e**. IF stainings of ALDH1 (red) were shown, and nuclei were counterstained with DAPI (blue), bar = 100 μm. **f**. Quantitation of ALDH1^+^ cells in control/Bevacizumab-treated/Bevacizumab + SHH002-hu1-treated tumors. Data were shown as mean ± SD (n = 5), ^*^p < 0.05, ^**^p < 0.01. **g**. SHH002-hu1 repressed the accumulation of β-catenin in the nucleus induced by Bevacizumab. IF stainings of β-catenin (red) were shown, and nuclei were counterstained with DAPI (blue), bar = 50 μm

The tumor photographs (Fig. 5a) and tumor growth curves (Fig. 5b) of each group showed that SHH002-hu1 inhibited the tumor growth in TNBC xenografts (MDA-MB-231: ^*^p < 0.05, MDA-MB-468: ^**^p < 0.01), and Bevacizumab + SHH002-hu1 significantly inhibited tumor growth when compared either to PBS control (MDA-MB-231: ^**^p < 0.01, MDA-MB-468: ^**^p < 0.01) or Bevacizumab alone treatment group (MDA-MB-231: ^**^p < 0.01, MDA-MB-468: ^**^p < 0.01). Let it be noted that Bevacizumab + SHH002-hu1 exhibited similar anti-proliferative activity with Bevacizumab + Docetaxel, which is one of the effective clinical therapies of TNBC. On account of that Bevacizumab is mostly combined with chemotherapy, therefore a combination of Bevacizumab + Docetaxel + SHH002-hu1 was tested to check whether this combination could further reduce or even fully remove the tumor burdon. The results showed that Bevacizumab + Docetaxel + SHH002-hu1 significantly inhibited tumor growth when compared either to PBS control (MDA-MB-231: ^**^p < 0.01, MDA-MB-468: ^**^p < 0.01) or Bevacizumab + Docetaxel group (MDA-MB-231: ^**^p < 0.01, MDA-MB-468: ^*^p < 0.05) (Supplementary Fig. 5).

Then, ALDH1-Hypoxyprobe double labeling of MDA-MB-231/MDA-MB-468 tumor tissues indicated that whereas tumors from control animals exhibited little hypoxia as determined by pimonidazole adduct (Hypoxyprobe) staining, tumors from Bevacizumab-treated mice displayed multiple areas of intense hypoxia. Further assessing the spatial relationship between ALDH1^+^ cells (ALDH1 has been identifed to be a functional marker for the epithelial-like CSCs derived from different cancer types) and areas of hypoxia within TNBC tumors, we observed high-density areas of ALDH1^+^ cells within hypoxic regions of tumors from Bevacizumab-treated mice, in contrast, ALDH1^+^ cells were scattered throughout tumors from control animals and the ALDH1^+^ staining was junior (Fig. 5c). Besides, the mesenchymal-like CSCs, strongly implicated in tumor invasion and resistance to therapy, are characterized by the CD44^+^ phenotype. Hence, the expression of CD44 was evaluated, and the results of CD44-Hypoxyprobe double labeling were similar to ALDH1-Hypoxyprobe double labeling (Supplementary Fig. 6A). Together, these findings confirmed that Bevacizumab stimulated the CSCs population by generating hypoxia within TNBC. Importantly, breast CSCs have been verified to link to tumor invasion and metastasis [17], probably accounting to the unsatisfactory therapeutic effects of Bevacizumab. As Bevacizumab activates Wnt/β-catenin pathway abnormally in the serum-deprived environment in vitro, we assessed the activity of Wnt/β-catenin pathway of TNBC tumor tissues in response to the inhibition of angiogenesis. Figure 5d illustrated that β-catenin was primarily detected in the cytoplasm of tumor cells from control mice. In contrast, cells within Bevacizumab-treated tumors displayed distinct nuclear localization of β-catenin, especially prominent in hypoxic areas. Hence, the increase in CSCs following Bevacizumab treatment is at least partly regulated by the Wnt/β-catenin signaling pathway.

In marked contrast to Bevacizumab treatment alone, the percentage of ALDH1^+^ cells of Bevacizumab + SHH002-hu1 group evidently decreased in MDA-MB-231/MDA-MB-468 tumor tissues (Fig. 5e). As shown in Fig. 5f, compared to Bevacizumab group, the percentage of ALDH1^+^ cells decreased 41.8% in MDA-MB-231 tumor tissues and 67.3% in MDA-MB-468 tumor tissues when treated by Bevacizumab + SHH002-hu1 (MDA-MB-231: ^*^p < 0.05, MDA-MB-468: ^**^p < 0.01). Similarly, compared to Bevacizumab group, the percentage of CD44^+^ cells decreased significantly in MDA-MB-231/MDA-MB-468 tumor tissues of Bevacizumab + SHH002-hu1 group (MDA-MB-231: ^**^p < 0.01, MDA-MB-468: ^**^p < 0.01) (Supplementary Fig. 6B, C). Further, when combined with Bevacizumab, SHH002-hu1 inhibited nuclear translocation of β-catenin and reduced the total expression level of β-catenin (Fig. 5g), which were in agreement with the data in vitro. In conclusion, SHH002-hu1 effectively reverses the increased subpopulation of putative TNBC stem-like cells induced by Bevacizumab-generating hypoxia via disrupting Wnt/β-catenin signaling pathway, indicating the potential of SHH002-hu1 to repress tumor cells invasion and metastasis, and attenuate the self-renew and tumor-initiating capacity of TNBC cells.

### SHH002-hu1 weakens the tumor-initiating capacity and self-renew capacity of TNBC cells enhanced by Bevacizumab

**Fig. 6:**
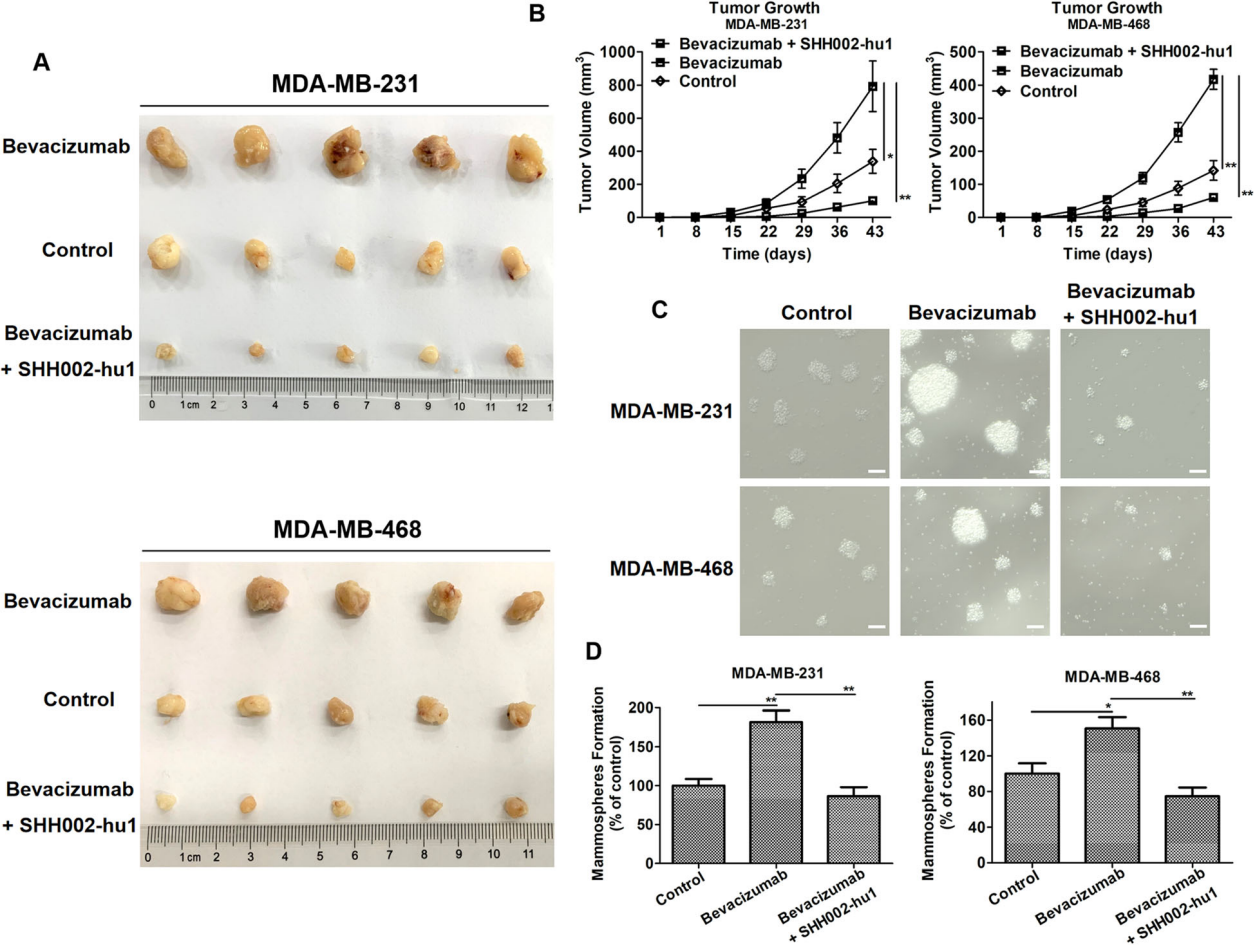
SHH002-hu1 weakens the ability of residual TNBC cells to initiate tumors and the self-renew capacity of TNBC cells enhanced by Bevacizumab. **a**. Representative images of isolated MDA-MB-231/MDA-MB-468 tumors replanted in secondary nude mice. **b**. Mice were implanted with tumor cells from control/Bevacizumab-treated/Bevacizumab + SHH002-hu1-treated mice. Each secondary nude mouse was inoculated with 1 × 10^4^ cells in one of the inguinal mammary fat pads. The growth of tumors was monitored weekly. Data were given as the mean ± SD (n = 5), ^*^p < 0.05, ^**^p < 0.01. **c**. 5 × 10^3^ cells isolated from MDA-MB-231/MDA-MB-468 xenografts were cultured in low attachment 6 well culture plates for 2 weeks to form the mammospheres, bar = 100 μm. **d**. Quantitation of the mammosphere-forming efficiency. The numbers of mammospheres (> 60 μm) were recorded after 2 weeks of culture. Data were presented as the mean ± SD, n = 3, ^*^p < 0.05, ^**^p < 0.01

SHH002-hu1 has been demonstrated to enhance the anti-tumor effects of Bevacizumab against TNBC tumor growth via impairing cancer stem-like properties. Then, secondary nude mouse xenograft model and sphere formation assay were utilized to investigated whether SHH002-hu1 holds the potential of reducing the risk of relapse and metastasis by impairing the self-renewal ability of TNBC cells. As shown in Fig. 6a, b, tumor cells isolated from Bevacizumab-treated mice exhibited significantly increased tumor-initiating capacity and growth in secondary mice compared with cells isolated from control tumors (MDA-MB-231: ^*^p < 0.05, MDA-MB-468: ^**^p < 0.01). Through analyzing the data of Bevacizumab + SHH002-hu1 group, we found that the ability of residual cancer cells from the combination groups to initiate tumors upon reimplantation in secondary mice was strongly inhibited compared to Bevacizumab group (MDA-MB-231: ^**^p < 0.01, MDA-MB-468: ^**^p < 0.01). Meanwhile, the dissociated tumor cells were produced from transplanted tumors of different groups for sphere formation assay. An obvious increase in mammosphere formation was observed from Bevacizumab group compared to the control, and SHH002-hu1 attenuated the mammosphere formation capacity of TNBC cells induced by Bevacizumab significantly (MDA-MB-231: ^**^p < 0.01, MDA-MB-468: ^**^p < 0.01) (Fig. 6c, d), which was consistent with the result of sphere formation assay using TNBC cells in vitro culture (Supplementary Fig. 7). Above all, SHH002-hu1 suppressed Bevacizumab-enhanced tumor-initiating and self-renew capacity of TNBC cells, predicting that SHH002-hu1 could prevent TNBC recurrence and metastasis caused by Bevacizumab.

### SHH002-hu1 reduces the adaptability of TNBC cells to hypoxia

In this study, Bevacizumab has been demonstrated to enhance cancer stem-like properties, tumor-initiating and self-renew capacity of TNBC cells via causing acute hypoxic stress. However, the limited efficacy of anti-angiogenesis drugs against TNBC is also thought to be in part due to angiogenesis rebound and some other adaptions to hypoxia through activation of genes that increase glycolysis and regulate pH. Moreover, cellular adaptions to low oxygen are principally regulated by the transcriptional activity of HIF-1 [18, 19]. Therefore, whether SHH002-hu1 exhibited the potency of impairing TNBC cells adaption to hypoxia was studied.

**Fig. 7:**
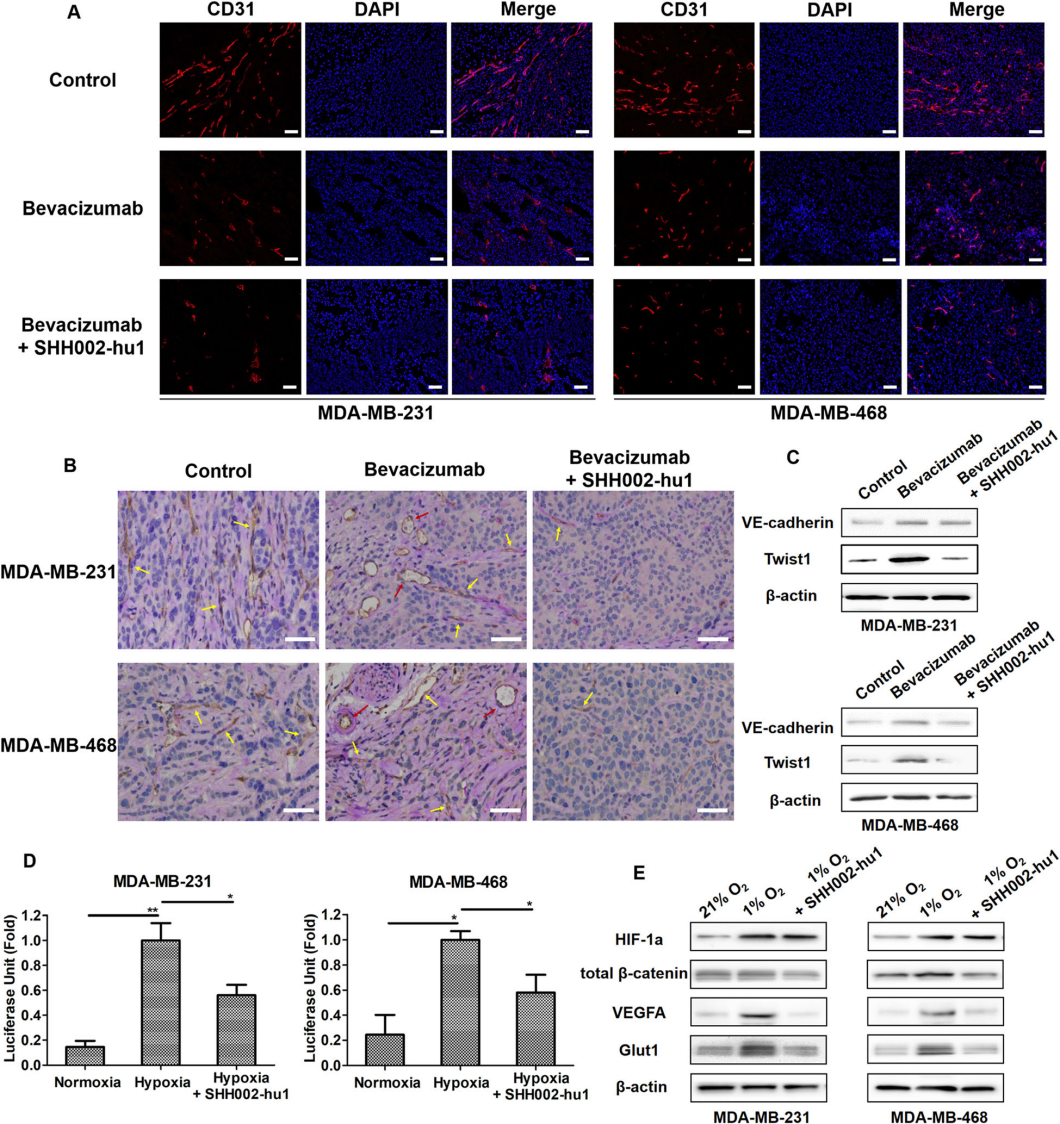
SHH002-hu1 reduces the adaptability of TNBC to hypoxia. **a**. IF assay of CD31 staining (red) indicated that SHH002-hu1 enhanced the anti-angiogenesis effect of Bevacizumab against TNBC, bar = 100 μm. **b**. IHC assay of PAS/CD31 double staining after drug discontinuation showed that the number of VM channels (red arrows) increased in the Bevacizumab group compared to the control, and the endothelium-dependent vessels (yellow arrows) rebounded after Bevacizumab discontinuation. SHH002-hu1 inhibited VM and the endothelium-dependent vessels rebound induced by Bevacizumab significantly. **c**. Western blot assay showed that the VM-associated proteins VE-cadherin and Twist1 were upregulated in the Bevacizumab-treated MDA-MB-231/MDA-MB-468 tumors, and SHH002-hu1 obviously suppressed the phenomenon. **d**. The HIF-1a transcriptional activity was enhanced by hypoxia remarkably, and SHH002-hu1 effectively inhibited the HIF-1a transcriptional activity under hypoxic condition. Data were presented as the mean ± SD, n = 3, ^*^p < 0.05, ^**^p < 0.01. **e**. Western blot assay showed that the expression of HIF-1a and HIF-1a targets (VEGFA, Glut1) was enhanced by hypoxia, and SHH001-hu1 could suppress the expression of VEGFA/Glut1 through inhibiting the expression of β-catenin under hypoxic condition

Bevacizumab/Bevacizumab + SHH002-hu1 was administered for 2 weeks and discontinued for a week, then the TNBC tumor-bearing nude mice were killed and the tumor tissues were collected for the follow-up detections. The CD31 staining assay of MDA-MB-231/MDA-MB-468 tumor tissues at the end of the administration indicated that Bevacizumab could effectively inhibit tumor angiogenesis compared to the PBS control (Fig. 7a). Nevertheless, IHC assay of PAS/CD31 double staining after drug discontinuation (Fig. 7b) showed that the number of VM channels increased in the Bevacizumab group compared to the control. VM channels are formed by tumor cells instead of endothelial cells, and connect with endothelium-dependent vessels to provide blood for tumor, which are responsible for the tumor angiogenesis rebound and treatment failure of anti-angiogenic agents in TNBC [20, 21]. It was comforting to find that SHH002-hu1 reduced the VM channels remarkably (Fig. 7b), illustrating the potential capacity of SHH002-hu1 to suppress TNBC tumor angiogenesis rebound when combined with Bevacizumab. The results of western blot assay (Fig. 7c) showed that the expression of VE-cadherin, a VM-associated molecule, was upregulated in the Bevacizumab-treated MDA-MB-231/MDA-MB-468 tumors compared with the control. Moreover, Bevacizumab increased Twist1 expression in the MDA-MB-231/MDA-MB-468 tumors. Previous studies have suggested that Twist1 upregulation can induce VE-cadherin expression in TNBC, and induce TNBC cells to generate more CSCs, which then promote VM channel formation in matrigel [22]. SHH002-hu1 was then be found to strongly reverse the increased expression of VM-associated critical proteins, VE-cadherin and Twist1, induced by Bevacizumab. As above, SHH002-hu1 could prevent angiogenesis rebound induced by Bevacizumab discontinuation through reducing VM channels.

To further elucidate the effects of SHH002-hu1 to HIF-1a and hypoxia adaption-associated proteins in hypoxia state, MDA-MB-231/MDA-MB-468 cells were grown under 21% O_2_ (normoxia) or 1% O_2_ (hypoxia, SHH002-hu1 was added in the group), then cells were collected for next detection. Figure 7d illustrated that HIF-1a transcriptional activity of MDA-MB-231/MDA-MB-468 cells was enhanced by hypoxia significantly, and SHH002-hu1 effectively inhibited the HIF-1a transcriptional activity under hypoxic condition. The results of western blot (Fig. 7e) showed that, hypoxia obviously increased the expression of HIF-1a, and the addition of SHH002-hu1 had no significant effect on the expression of HIF-1a. There was no significant difference in total β-catenin expression level between normoxia and hypoxia, while SHH002-hu1 reduced the expression of total β-catenin. The β-catenin pathway has been reported to interact with HIF proteins in multiple ways [23]. In normoxia, the constitutive β-catenin functions as a major coactivator of TCF-LEF activity in the Wnt pathway; in hypoxia, constitutive β-catenin can be rapidly switched to enhance HIF-1a mediated transcription and promote cell survival [24]. Hence, the reduced expression of total β-catenin probably accounted for why SHH002-hu1 effectively inhibited the HIF-1a transcriptional activity without affecting the expression of HIF-1a. Furthermore, the expression detection of HIF-1a classical targets revealed that, SHH002-hu1 remarkably inhibited the expression of VEGFA and Glut1 increased by hypoxia. In conclusion, SHH002-hu1 reduces the adaptability of TNBC cells to hypoxia via suppressing HIF-1a transcriptional activity and the expression of hypoxia adaption-associated proteins.

## Discussion

With the rapid development of immunotherapy and antibody-drug conjugate (ADC), the clinical therapeutics of TNBC are heralding the dawn of a new era full of hope. The antibody targeting programmed cell death 1 ligand-1 (PD-L1) Tecentriq in combination with Abraxane brought significant benefit in PFS to TNBC patients; hence, FDA accelerated the approval of the combination for the first-line treatment of PD-L1^+^ TNBC on 8 March, 2019 [25]. Sacituzumab govitecan (IMMU-132) is a novel ADC in which SN-38, a topoisomerase I inhibitor, is coupled to the humanized anti-trophoblast cell-surface antigen 2 (Trop-2) monoclonal antibody. The phase III ASCENT study of IMMU-132 for the treatment of metastatic TNBC has been brought to an early end, benefiting from the superior efficacy, and FDA accelerated the approval of IMMU-132 for the treatment of patients with metastatic TNBC who have received at least two previous therapies [26]. However, for PD-L1^−^ TNBC and triple-negative operable primary invasive breast cancer, it is imperative to explore novel therapeutic options to improve the therapeutic effectiveness.

Inhibiting angiogenesis is demonstrated a potential strategy for the treatment of TNBC, as genes involved in angiogenesis are frequently activated in basal-like tumors [27]. Bevacizumab has exhibited clinical efficacy in combination with chemotherapy in patients with HER2-negative metastatic breast cancer [28], including in subgroups of patients with metastatic TNBC [29, 30]. Indeed, early adoption of Bevacizumab could maximise its benefit, because trapping of VEGFA, would be most effective in the early carcinogenesis niche where only a few angiogenic factors exist [31]. The addition of Bevacizumab to new adjuvant chemotherapy treatment (NACT) increased the pathologic complete response (pCR) rates, but the results of improving relapse-free or OS failed to meet expectations (CALGB 40603 (Alliance)) [32]. A series of relevant studied indicate that the abnormally activated Wnt/β-catenin signaling pathway of Bevacizumab-treated TNBC in response to hypoxia possibly accounts for the undesired outcomes [33–35], calling for the combination of Bevacizumab and Wnt/β-catenin signaling targeting drugs.

The Wnt/β-catenin signaling pathway is a significant pathway that regulates cell proliferation, migration, and differentiation, thus making it a powerful regulator of embryonic development and tumorigenesis [36]. Recent studies indicate that Wnt/β-catenin signaling is particularly over-activated in TNBC [37], and the overexpression of Fzd7, a key receptor protein of Wnt/β-catenin signaling, is observed in TNBC [13]. Aberrations in Wnt signaling have been shown to cooperate with other signaling pathways, oncogenes and tumour suppressors. Owing to these complexities and the potential redundancy of many pathway components, direct targeting of Wnt signaling has been difficult [38]. The context-specific activity and redundancy found in the receptor functions suggest that selective targeting of Wnt receptors responsible for disease phenotypes is possible, without greatly disrupting normal tissue homeostasis [39]. Given the important roles Fzd7 played in tumorigenesis and progression, so far, several methods have been designed to antagonize Wnt/β-catenin signaling by targeting Fzd7 [40], among which, the more translatable method was the utilization of antibodies to target Fzd7. A monoclonal antibody (vantictumab, OMP-18R5) targeting Fzd7 has been developed by OncoMed Pharmaceuticals, Inc. [41]. The phase I trial (NCT01973309) of metastatic breast cancer has showed that OMP-18R5 inhibited the growth of breast cancer and reduced the frequency of tumor-initiating cells in combination with pacilitaxel. While OMP-18R5, having cross-reactivity with 4 other Fzd receptors (Fzd1/2/5/8), may show broad application for a wide range of cancers, the lack of specificity towards its intended target poses concerns for its off-target effects.

In this study, we utilized hybridoma technique and antibody humanization technique to generate a high-affinity antibody specifically targeting Fzd7. Fzd7 protein contains a large extracellular N-terminal cysteine-rich domains (CRD) that provides a surface for specific Wnt binding. Hence, the recombinant human Fzd7 extracellular CRD protein was used to immunize mice to obtain the mouse monoclonal antibody (SHH002). Because of the important role of ligand binding, the sequences in CRD are highly conserved between Fzds within and across species. CRD of the Fzd7 receptor share over 40% sequence identity with other members of Fzd family, especially Fzd1/2/5/8. As a result, the antibodies generated by immunizing mice with recombinant Fzd7 CRD protein were likely to bind with other members of Fzd family, including OMP-18R5. To screen out the antibodies specifically bind with Fzd7, ELISA assay was utilized to evaluate the binding of the mouse antibodies with Fzd1/2/5/8 (data were not shown), and SHH002 was picked out with no cross-reactivity with Fzd1/2/5/8. Then, the Human Germline sequences library and Discoverystudio software were adopted to complete the humanized design of SHH002, the humanization degree of the final humanized antibody SHH002-hu1 reached 98%. The specificity of SHH002-hu1 was further confirmed by BLI. Therefore, the generated homologue-specific anti-Fzd7 antibody would serve a dual role as an affinity reagent for the investigation of context-specific functions for Fzd7, and also as a potential therapeutic agent that may be used alone or in combination with the anti-angiogenesis drugs.

A series of studies in vitro and in vivo indicated that SHH002-hu1 could effectively target Fzd7^+^ cells (Fig. 2a-c) and Fzd7^+^ TNBC tumor tissue (Fig. 2d, e), and inhibit TNBC via blocking Wnt/β-catenin signaling pathway (Fig. 3d-f). When combined with Bevacizumab, SHH002-hu1 significantly enhanced the capacity of Bevacizumab in inhibition of MDA-MB-231/MDA-MB-468 tumor growth (Fig. 5a, b). To dig deep into the mechanism involved, we found that SHH002-hu1 reversed the increased subpopulation of putative TNBC stem-like cells induced by Bevacizumab-generating hypoxia via disrupting Wnt/β-catenin signaling pathway (Fig. 5e-g), indicating the potential of SHH002-hu1 to repress TNBC cells invasion and metastasis. In addition, SHH002-hu1 attenuated Bevacizumab-enhanced tumor-initiating and self-renew capacity of TNBC cells (Fig. 6), illustrating that SHH002-hu1 shows the potential of preventing TNBC recurrence and metastasis when combined with Bevacizumab. Furthermore, SHH002-hu1 prevented angiogenesis rebound induced by Bevacizumab through reducing VM channels, and suppressed HIF-1a transcriptional activity and the expression of hypoxia adaption-associated proteins, revealing the potency of SHH002-hu1 to impair TNBC adaption to hypoxia (Fig. 7).

## Conclusions

In summary, SHH002-hu1, a novel humanized antibody specifically targeting Fzd7, was successfully generated in this study. SHH002-hu1 is sufficient to inhibit Bevacizumab-induced cancer stem-like properties and EMT via abating Wnt/β-catenin signaling. SHH002-hu1 shows the potential of improving the treatment efficacy of Bevacizumab, further reduces the risk of TNBC recurrence and metastasis. Hence, the combination regimen of Bevacizumab and SHH002-hu1 provides an effective candidate for the clinical treatment of TNBC, especially PD-L1^−^ TNBC and triple-negative operable primary invasive breast cancer.

## Supplementary Information

**Additional file 1.**

**Additional file 2.**

**Additional file 3.**

**Additional file 4.**

**Additional file 5.**

**Additional file 6.**

**Additional file 7.**

**Additional file 8.**

**Additional file 9.**

## Data Availability

All remaining data are available within the article and supplementary files, or available from the authors upon request.

## Abbreviations

ADC: Antibody-drug conjugate
AV: Annexin V
BLI: Biolayer interferometry
CRD: Cysteine-rich domains
CSCs: Cancer stem cells
ECGS: Endothelial cell growth supplement
ECM: Endothelial culture medium
EGF: Epidermal growth factor
ER: Estrogen receptor
EMT: Epithelial-mesenchymal cell transformation
FGF: Fibroblast growth factor
Fzd7: Frizzled-7
HC: Heavy Chain
HER2: Human epidermal growth factor receptor 2
HIF-1a: hypoxia-inducible factor-1α
IF: Immunofluorescence
IHC: Immunohistochemistry
LC: Light Chain
mCRC: Metastatic colorectal cancer
NACT: New adjuvant chemotherapy treatment
NIR: Near infrared
NSCLC: Non-small cell lung cancer
ORR: Objective response rate
OS: Overall survival
pCR: Pathologic complete response
PD-L1: Programmed cell death 1 ligand-1
PFS: Progression-free survival
PI: Propidium iodide
PR: Progesterone receptor
ROI: Region of interests
SD: Standard deviation
TNBC: Triple-negative breast cancer
Trop-2: Trophoblast cell-surface antigen 2
VEGFA: Vascular endothelial growth factor A
VM: Vasculogenic mimicry
