# Supplementary figures and images for: A novel humanized Frizzled-7-targeting antibody enhances antitumor effects of Bevacizumab against triple-negative breast cancer via blocking Wnt/β-catenin signaling pathway
Source: J Exp Clin Cancer Res. 2021 Jan 12;40:30. doi: 10.1186/s13046-020-01800-x (PMC7802198; doi:10.1186/s13046-020-01800-x)

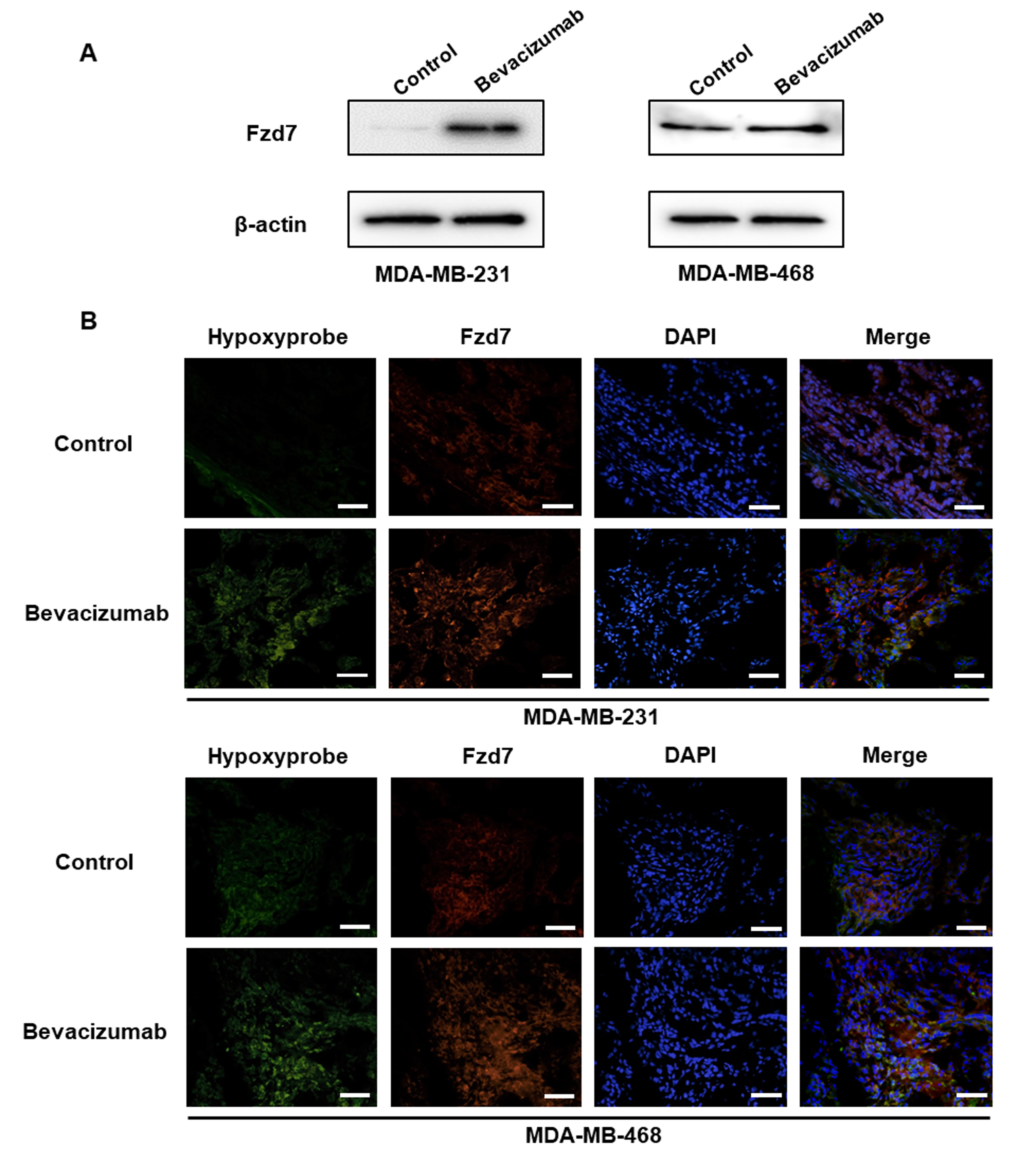

Supplement: Supplementary file 1 — Additional file 1. [file 13046_2020_1800_MOESM1_ESM.tif]

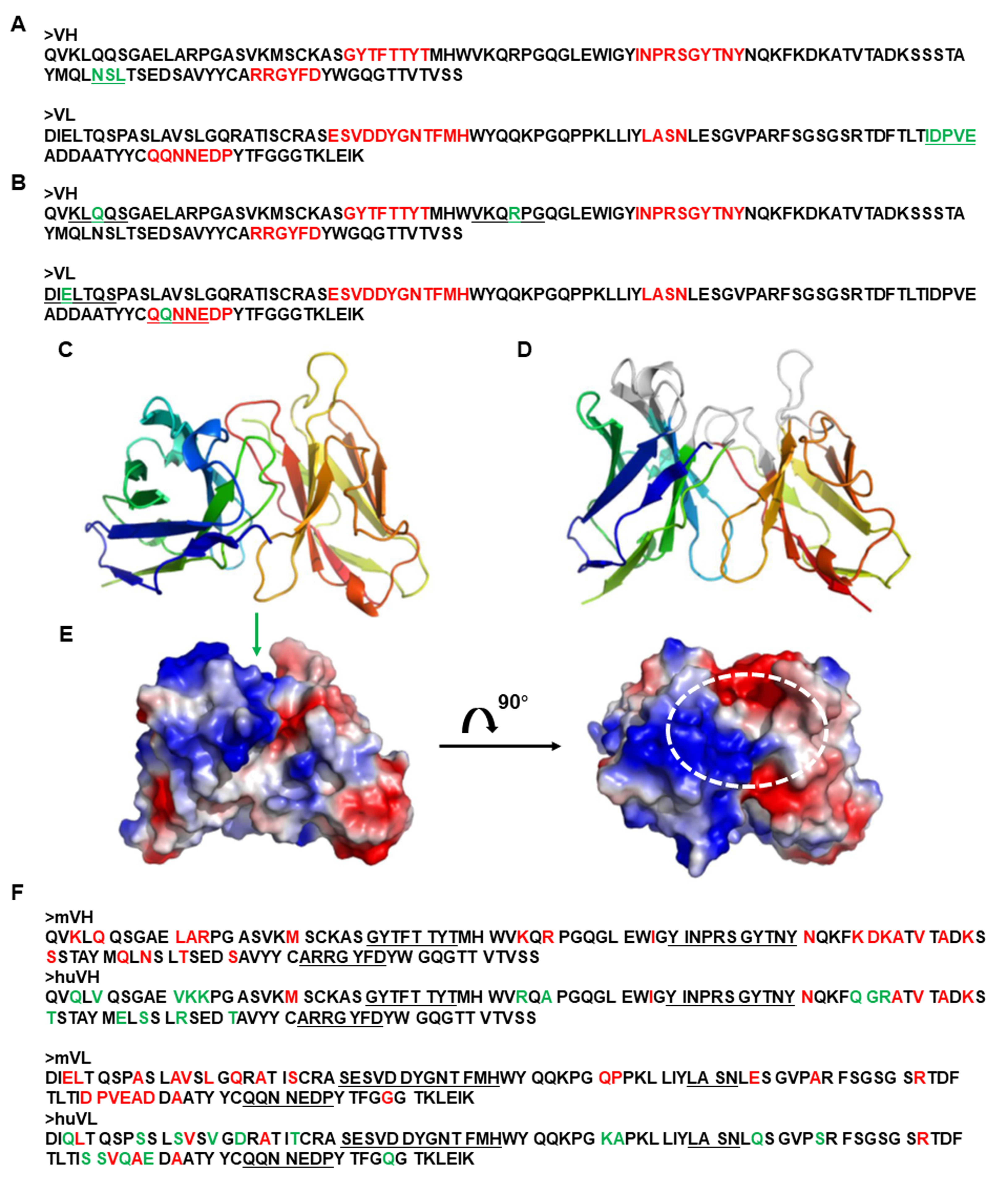

Supplement: Supplementary file 2 — Additional file 2. [file 13046_2020_1800_MOESM2_ESM.tif]

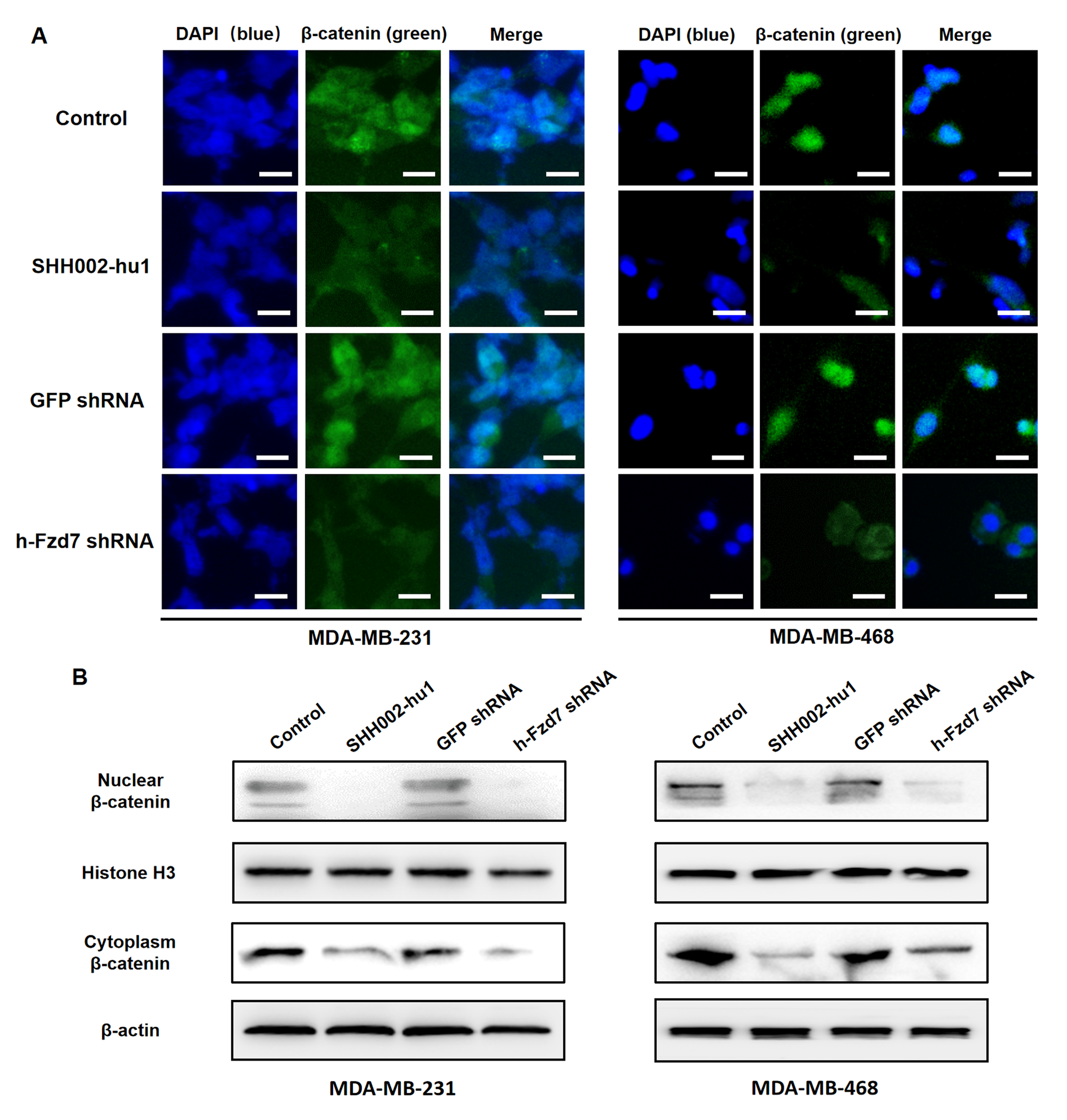

Supplement: Supplementary file 3 — Additional file 3. [file 13046_2020_1800_MOESM3_ESM.tif]

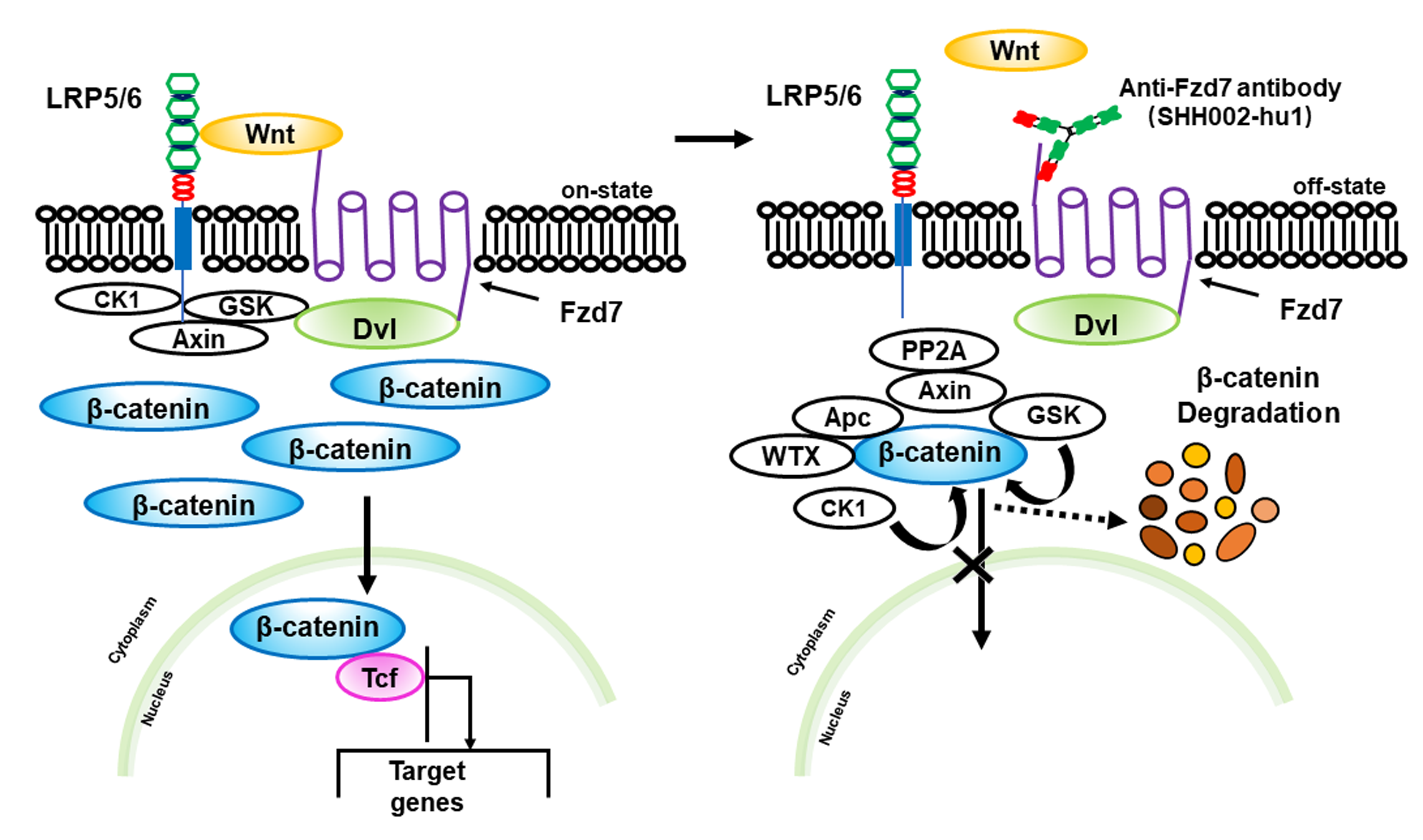

Supplement: Supplementary file 4 — Additional file 4. [file 13046_2020_1800_MOESM4_ESM.tif]

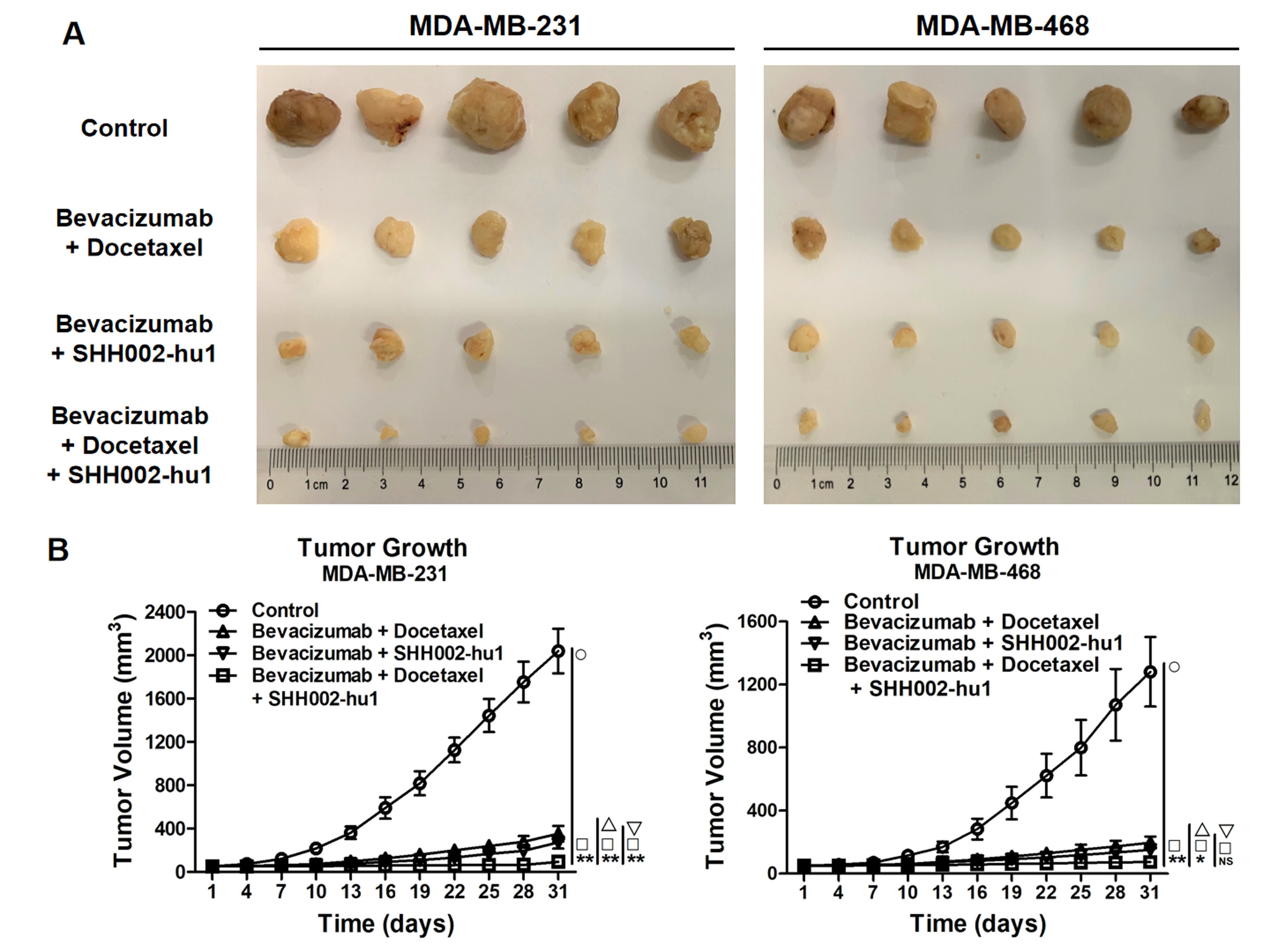

Supplement: Supplementary file 5 — Additional file 5. [file 13046_2020_1800_MOESM5_ESM.tif]

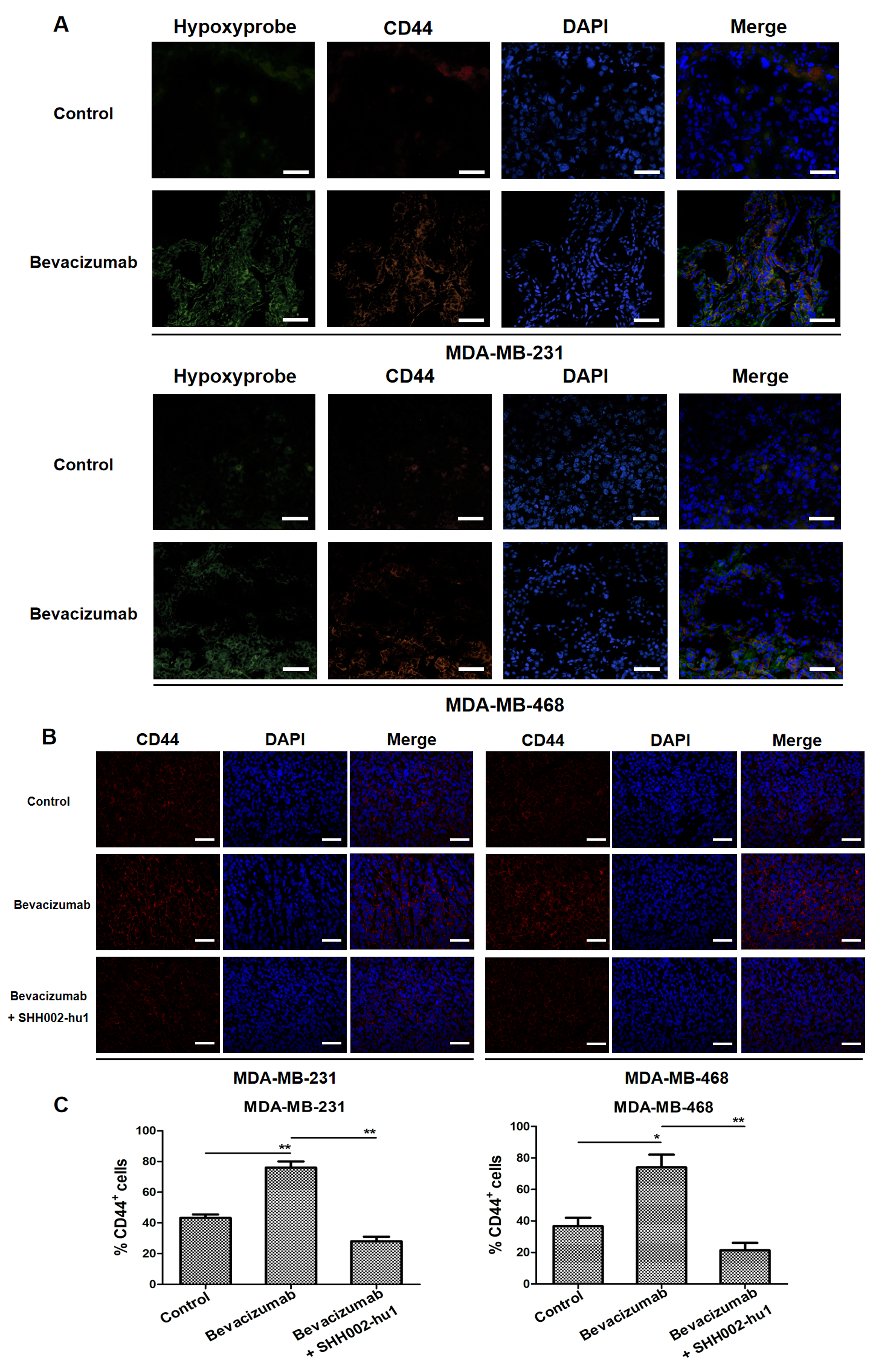

Supplement: Supplementary file 6 — Additional file 6. [file 13046_2020_1800_MOESM6_ESM.tif]

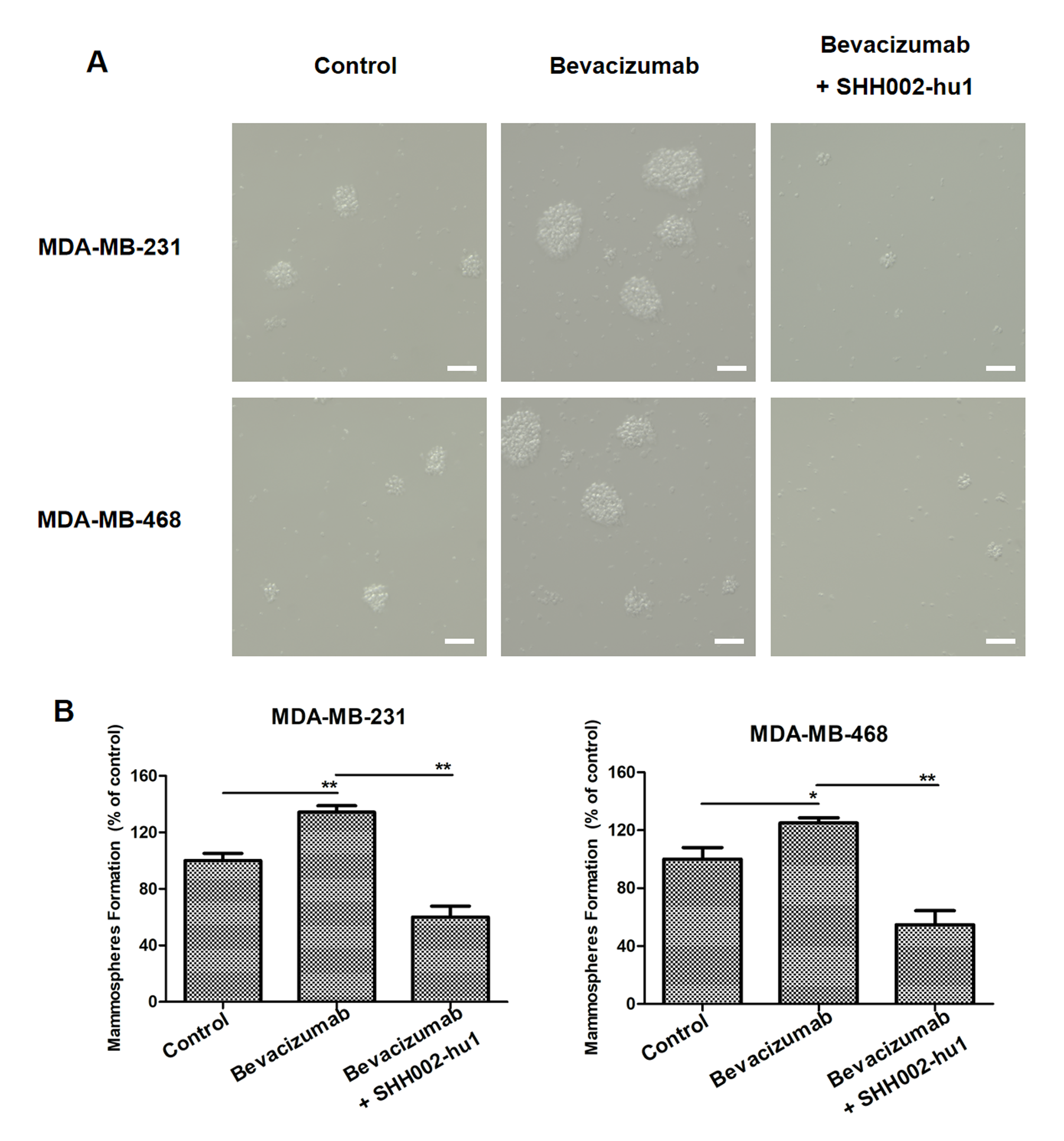

Supplement: Supplementary file 7 — Additional file 7. [file 13046_2020_1800_MOESM7_ESM.tif]
