# Supplementary material for: A novel humanized Frizzled-7-targeting antibody enhances antitumor effects of Bevacizumab against triple-negative breast cancer via blocking Wnt/β-catenin signaling pathway
Source: J Exp Clin Cancer Res. 2021 Jan 12;40:30. doi: 10.1186/s13046-020-01800-x (PMC7802198; doi:10.1186/s13046-020-01800-x)
Supplement: Supplementary file 8 — Additional file 8. [file 13046_2020_1800_MOESM8_ESM.pdf]

Supplementary Materials and Methods

Detection of Fzd7 expression in Bevacizumab-treated TNBC cells and tumor tissues

MDA-MB-231/MDA-MB-468 cells were treated with Bevacizumab (200 nmol/L) for 24 h under serum deprivation condition, then the whole cell proteins were extracted. Western blots were probed with α-Fzd7 (Abcam, USA). The xenograft tumors of MDA-MB-231/MDA-MB-468 cells in nude mice was established. When the average tumor volume reached 50 mm^3, mice were randomized into 2 groups (n = 5 for each group), and the administration began: (1) PBS control; (2) 5 mg/kg Bevacizumab (intravenous injection, twice a week). For Fzd7-Hypoxyprobe double labeling, mice were injected intravenously with 60 mg/kg of the pimonidazole solution, 90 min later, the mice were euthanatized and tumor tissues were then removed and snap-frozen. Frozen tissue sections were then interrogated with FITC-conjugated α-pimonidazole and α-Fzd7 followed by respective Cy3-conjugated secondary IgG. Coverslips were then mounted with DAPI stain solution.

Humanized design of SHH002

SHH002 is a murine monoclonal antibody targeting Frizzled-7 obtained by our lab through the hybridoma technology, SHH002 exhibited high affinity with rhFzd7 (KD

< 1.0 × 10^{-12} M), the subtype of heavy chain is IgG2b, the subtype of light chain is Kappa. The template of humanized design was determined after the comparison of variable region sequences of SHH002 with Human Germline sequences library. Then, the Discoverystudio software was utilized to complete the humanized design of SHH002, mouse-derived amino acids critical to the structural stability of antibodies were retained, and the amino acid residues of non-critical sites were mutated into human amino acids.

Silencing the expression of Fzd7

The shRNA of Fzd7 (h-Fzd7 shRNA, Top strand: GATCCGCGCTCATGAACAAGTTCGGCTTCCATTCAAGAGATGGAAGCCGAACTTGTTCATGAGCGTTTTTTG; Bottom strand: AATTCAAAAAACGCTCATGAACAAGTTCGGCTTCCATCTCTTGAATGGAAGCCGAACTTGTTCATGAGCGCG) was designed, and the lentiviral vector pHBLV-U6-MCS-CMV-ZsGreen-PGK-PURO was used. Then, h-Fzd7 shRNA was used to disrupt the expression of Fzd7, and h-Fzd7 shRNA-virus-transduced TNBC cells were set as a positive control in the IF and Western blot assays of the expression and localization of β-catenin.

Sphere formation assay

MDA-MB-231/MDA-MB-468 cells were treated by Bevacixumab (200 nmol/L)/Bevacixumab (200 nmol/L) + SHH002-hu1 (100 nmol/L) for 48 h, then 5×10^3 dissociated MDA-MB-231/MDA-MB-468 cells were seeded on ultralow attachment 6-well plates in serum-free medium DMEM/F12 (Gibco, Grand Island, USA) supplemented with B27 (Gibco, Grand Island, USA), 20 ng/mL human recombinant fibroblast growth factor (FGF), and 20 ng/mL epidermal growth factor (EGF, Sino Biological Inc., Beijing, China). The mammospheres (diameter > 60 μm) were counted under an OLYMPUS inversion fluorescence microscope.
