# Supplementary material for: A novel humanized Frizzled-7-targeting antibody enhances antitumor effects of Bevacizumab against triple-negative breast cancer via blocking Wnt/β-catenin signaling pathway
Source: J Exp Clin Cancer Res. 2021 Jan 12;40:30. doi: 10.1186/s13046-020-01800-x (PMC7802198; doi:10.1186/s13046-020-01800-x)
Supplement: Supplementary file 9 — Additional file 9. [file 13046_2020_1800_MOESM9_ESM.pdf]

Supplementary Figure legends

Supplementary Figure 1. Bevacizumab treatment up-regulated the expression of Fzd7 in TNBC. A. Bevacizumab increased the expression of Fzd7 in MDA-MB-231/MDA-MB-468 cells under serum deprivation condition. The results of Western blot analysis for the Fzd7 expression were shown, β-actin was used as loading control. B. Bevacizumab treatment increased the expression of Fzd7 in MDA-MB-231/MDA-MB-468 tumor tissues. Staining showed pimonidazole immunodetection (green) and Fzd7 (red) merged with DAPI-stained nuclei (blue), bar = 100 μm. Compared to the control, TNBC tumor tissues from Bevacizumab-treated mice displayed significantly more areas of intense hypoxia and more expression of Fzd7, and Fzd7 positivity was found specific to zones of low oxygen.

Supplementary Figure 2. The humanized design of SHH002. A. The analysis results of the potential modification sites (marked in green) in the VH/VL sequence of SHH002. The sequences marked in red represented CDR. B. The immunogenicity analysis predicted that there were some amino acid sequences with high immunogenicity (marked with underline) in VH/VL of SHH002. The amino acids marked in green represented the hot spots. C. The structure simulation graph of VH and VL (SHH002) from Discoverystudio software, the reliability was 98%. The left portion of software simulating graph indicated the VH of SHH002, and the right portion indicated the VL. D. The gray areas in the software simulating graph indicated the CDR of SHH002. E. The structure simulation graph of VH/VL surface charge from Discoverystudio software. The red areas indicated negative charge, and the blue areas indicated positive charge. The green arrow pointed to CDR in the left hand picture, and the area in white oval frame indicated CDR in the right hand picture. F. The variable region sequence of murine antibody (mVH and mVL) and the humanized antibody (huVH and huVL). The amino acids marked in red represented the mouse sequences, and the amino acids marked in green represented the mutational sites. The amino acid sequences marked with underline represented CDR.

Supplementary Figure 3. SHH002-hu1 significantly inhibited Wnt/β-catenin pathway. A. IF staining of β-catenin (green). β-catenin was detected in the nucleus in control and GFPshRNA-transduced MDA-MB-231/MDA-MB-468 cells. SHH002-hu1 and h-Fzd7 shRNA repressed the accumulation of β-catenin in the nucleus. Nuclei were counterstained with DAPI (blue). bar = 20 μm. B. Western blot of β-catenin. SHH002-hu1 and h-Fzd7 shRNA reduced nuclear accumulation of β-catenin remarkably. Histone H3 was used as loading control for nuclear proteins, and β-actin was for cytoplasmic proteins.

Supplementary Figure 4. Schematic representation of a model wherein anti-Fzd7 antibody inhibits Wnt/β-catenin signaling pathway. The anti-Fzd7 antibody blocks the ability of Wnt proteins to interact with Fzd7. Then, the formation of "destruction complex" induces the degradation of β-catenin, resulting in the inhibition of the Wnt/β-catenin signaling pathway.

Supplementary Figure 5. SHH002-hu1 enhanced the anti-tumor effect of Bevacizumab + Docetaxel. A. Representative images of isolated tumors from MDA-MB-231 (the left 5 rows)/MDA-MB-468 (the right 5 rows)-tumor bearing nude mice. B. MDA-MB-231/MDA-MB-468 tumor growth curves of each group under different treatments. Data were given as the mean ± SD (n = 5), $^{*}p < 0.05$, $^{**}p < 0.01$, NS: no significance.

Supplementary Figure 6. SHH002-hu1 decreased the percentage of $ALDH1^{+}$ cells increased by Bevacizumab. A. Bevacizumab induced hypoxia in TNBC tumor tissues

in vivo, and $CD44^+$ cells were concentrated in hypoxic regions. Hypoxia in MDA-MB-231/MDA-MB-468 tumors was detected by IF staining of pimonidazole adducts in sections from different groups. Staining showed pimonidazole immunodetection (green) and CD44 (red) merged with DAPI-stained nuclei (blue), bar = 100 μm. B. IF stainings of CD44 (red) were shown, and nuclei were counterstained with DAPI (blue), bar = 100 μm. C. Quantitation of $CD44^+$ cells in control/Bevacizumab-treated/Bevacizumab + SHH002-hu1-treated tumors. Data were shown as mean ± SD (n = 5), $^*p < 0.05$, $^{**}p < 0.01$.

Supplementary Figure 7. SHH002-hu1 remarkably attenuated the mammosphere formation capacity of TNBC cells induced by Bevacizumab. A. 5×10^3 MDA-MB-231/MDA-MB-468 cells cultured *in vitro* from different groups (control/Bevacixumab/Bevacixumab + SHH002-hu1) were cultured in low attachment 6 well culture plates for 2 weeks to form the mammospheres, bar = 100 μm. B. Quantitation of the mammosphere formation assay. The numbers of mammospheres (> 60 μm) were recorded after 2 weeks of culture. Data were presented as the mean ± SD, n = 3, $^*p < 0.05$, $^{**}p < 0.01$.
